# Changing lives, dynamic plans: Prospective assessment of 12-month changes in pregnancy timing intentions and personal circumstances using data from HER Salt Lake

**DOI:** 10.1371/journal.pone.0257411

**Published:** 2021-09-20

**Authors:** C. Geist, B. G. Everett, R. G. Simmons, J. N. Sanders, L. M. Gawron, K. Myers, D. K. Turok

**Affiliations:** 1 Department of Sociology and Division of Gender Studies, University of Utah, Salt Lake City, Utah; 2 Department of Obstetrics & Gynecology, University of Utah, Salt Lake City, Utah; University of Michigan, UNITED STATES

## Abstract

**Objectives:**

To explore the association between changes in personal circumstances and shifts in pregnancy intentions.

**Study design:**

New start contraceptive clients, who desired to prevent pregnancy for at least one year enrolled in the survey arm of the HER Salt Lake Contraceptive Initiative (September 2015 –March 2017) and responded to the question “What are your future pregnancy plans?” at enrollment and 12-month follow-up. We estimated multivariable binary logistic fixed-effects regressions to examine the association between changes in personal circumstances and a change from never desiring a pregnancy at enrollment to considering one in the future at 12-month follow-up.

**Results:**

The majority of the 2825 participants (2246, 79%) maintained their pregnancy timing intention over the 12-month study period. Multivariable analyses of the 208 participants who changed from never desiring a pregnancy to considering pregnancy in the future at 12-month follow-up indicated that entering cohabitation (aOR 3.14, 95% CI 1.30–7.58), increased household income (aOR 1.06, 95% CI 1.00–1.13), and changes from unemployment to full-time employment (aOR 5.94, 95% CI 1.29–27.36) are associated with increased the odds of desiring a future pregnancy after never wanting one a year prior.

**Conclusions:**

Pregnancy intentions are dynamic over twelve months and covary with partner status, household income, and employment status. Pregnancy intentions are linked to changes in life circumstances. Health care providers need to frequently assess pregnancy intentions and resulting contraceptive or preconception needs.

## Introduction

Pregnancy and parenting goals factor into contraceptive decision-making [1,2]. Clinicians who provide contraception can increase person-centered family planning care by incorporating reproductive goal conversations into contraception counseling [3,4]. From both a clinical and population health perspective, questions about desired timing of pregnancy hold relevance. These questions are often used to assess a client’s reproductive health needs and direct counseling on contraception or preconception care. For example, the PATH framework is a patient-centered reproductive goals counseling approach and can be used clinically to discuss Parenting and Pregnancy Attitudes, Timing, and How important it is to prevent pregnancy now [3,5,6]. One Key Question is even more specifically focused on the one-year time frame for pregnancy desire [7]. A prospective assessment of pregnancy timing intentions also reduces the bias resulting from asking whether a pregnancy was planned post-birth.

Desired family size and pregnancy timing intentions shift over the life course as individuals’ circumstances change [8–12]. The availability of a suitable partner or stable relationship is often seen as a desirable prerequisite for a planned pregnancy [8,13]. Pregnancy and parenthood involve substantial direct and indirect costs [14], and desired timing of pregnancy may fluctuate based on perceived ability to meet these costs. Unemployment, for example, has been associated with smaller ideal family size [15], and young people with low income or strained economic resources may opt to delay their parenthood (i.e., spend more time saving or improving their financial situation before having children) [16]. Enrollment in higher education or a lack of health insurance may also be perceived as at odds with a planned entry into parenthood [17]. However, there is also evidence that economic and social marginalization is associated with earlier entry into planned and unplanned parenthood [18]. Research on the link between dynamic sexuality and pregnancy desires is slowly emerging [19].

Deciding when to start or grow a family is deeply personal and complex. Understanding the multitude of factors that influence shifting attitudes about pregnancy and the desired timing of pregnancies can support providers in being flexible and responsive to contraceptive clients’ needs. Understanding the extent to which pregnancy intentions can change during a one-year period contributes to current efforts to de-stigmatize and support the common practices of contraceptive method switching and discontinuation [20]. Provider reluctance for “early” IUD and implant removal and focus on these methods’ cost-effectiveness after two years of use may be particularly problematic for individuals with dynamic pregnancy intentions or those who may desire pregnancy sooner than they initially thought when seeking their new method [21–24]. Although studies have identified potential factors that influence pregnancy decisions, few studies have assessed how these decisions may change or shift over time and which personal circumstances are associated with reproductive goal changes.

This study has two aims: First, we describe stability and change in pregnancy timing intentions over a 12-month period among a large, prospective cohort of reproductive-aged individuals who received no-cost contraception through a county-wide contraceptive initiative. Second, we use multivariable analyses to understand the association between changes in social and economic circumstances and intentions to desire a future pregnancy after previously stating to never desire one.

## Materials and methods

### Data

We used data from the HER Salt Lake Contraceptive Initiative. HER Salt Lake was a prospective cohort study that recruited participants from four family planning clinics in Salt Lake County, Utah (September 2015-March 2017). Over the 18-month enrollment period, a total of 11,509 individuals presented to participating family planning clinics to receive new contraceptive services or change their methods. Permanent contraception such as partner vasectomy or tubal ligation was not offered as part of the study. The questionnaire was beta tested prior to implementation for comprehension in English and Spanish. While we did not validate the questionnaire, we did use the same data collection instruments in a prior study in a much smaller population to assure that participants understood the content and could respond appropriately. We included the 12-month follow up survey questionnaire in the supplemental materials (S1 Codebook).

Of eligible clients, 38% enrolled in the longitudinal survey-arm (n = 4,425 for all enrollment periods combined). The 18-month enrollment phase began with a six-month control period, during which clients received standard of care and navigated existing payment structures. It was followed by 12 months of enrollment for the intervention, which began in March 2016. During this time, eligible clients received no-cost contraception of their choice, including the option to switch contraceptive methods for a period of three years. We describe the initial design and interventions in much greater detail elsewhere [25].

For the study’s survey-arm, eligible contraceptive clients included new and existing clients beginning a new contraceptive method, including those switching methods, aged 18 to 45, who desired to prevent pregnancy for at least one year. Additional eligibility criteria included fluency in English or Spanish, having a functioning phone number, and willingness to complete nine surveys over three years. Study staff completed the informed consent process with participants prior to enrollment. The current study uses baseline data from participants who entered the study during the 18-month enrollment phase and provided 12-month follow-up surveys. The University of Utah IRB approved all aspects of the study, including the consent procedure.

### Sample

This analysis includes 2,825 participants who comprise the “pregnancy intention sample.” These individuals provided pregnancy timing intention data at both the enrollment survey and the 12-month follow-up survey.

Our outcome of interest was pregnancy timing intentions, measured by the question: “What are your future pregnancy plans?” Response options included: 1) “I am currently trying to get pregnant” (only available at 12-month follow-up), “I would like to get pregnant in the next year,” 2) “I would like to get pregnant in the next 2–5 years, but not in the next year”, 3) “I would like to get pregnant in the next 5–10 years, but not before then”, 4) “I am uncertain if or when I would like to become pregnant (only at enrollment),” and 5) “I do not plan on getting pregnant at any time in the future.” Participants could also select “other”.

Of the 4,425 participants who enrolled, 87% (n = 3,837) participated in the 12-month follow-up. We excluded 747 participants who declared “uncertain” pregnancy intention timing. Our analysis sought to understand either stability or change in pregnancy timing intentions, and uncertainty was only fully assessed at enrollment. Including this large group would have artificially inflated reported change in pregnancy timing intentions. As the contraceptive needs of individuals with uncertain pregnancy intentions are particularly difficult to identify, we will examine them more closely in future research. We further excluded 44 participants who either did not answer the pregnancy timing intention question at enrollment (n = 31) or who selected “other” (n = 13). Among the remaining participants, 113 participants reported positive urine pregnancy tests (PUPT) between enrollment and 12-month follow-up. Of these, 37 indicated the PUPT was the result of trying to become pregnant. These individuals were classified as desiring a pregnancy “now” at the time of the follow-up survey, regardless of the exact date when their pregnancy started. We excluded 76 participants who indicated they had an unintended pregnancy between the two waves or who had a positive pregnancy test, and it was unclear whether the pregnancy was intended or not. We were concerned about our ability to accurately ascertain change in pregnancy intentions at the 12-month follow-up for this group. This group is not too dissimilar from the analytic sample (Table 1). However, Hispanic, sexual minority participants seem overrepresented (Table C in S1 Table) We excluded an additional 145 participants due to missing pregnancy intentions at the 12-month follow-up.

**Table 1:** Characteristics of HER Salt Lake participant with information on pregnancy intentions at enrollment and 12-month follow-up.

Variable	N at enrollment/12-month follow-up/both waves)	Mean/Proportion at enrollment	Mean/Prop. at 12 months follow-up	N/% of participants with change between waves
**Social Circumstances**				
*Age at enrollment (in years)* ^1^	2825/2825/2825	25	25	
*Race/Ethnicity* ^1^	2809/2809/2809			
White, non-Hispanic		64%	64%	
Hispanic (any racial group)		25%	25%	
Non-white, non-Hispanic/Latinx		11%	11%	
*Sexual identity*	2776/2688/2673			492 (18%)
Exclusively heterosexual		76%	68%	
Mostly heterosexual		13%	19%	
Mostly or exclusively gay/lesbian, bisexual, or other sexuality		11%	14%	
*Relationship status*	2769/2816/2760			1187 (43%)
Married		12%	19%	
Living together with a partner		49%	51%	
Actively dating		18%	10%	
Single (or divorced), not dating		21%	20%	
*Educational enrollment and goals*	2241/2401/2003			761 (38%)
Not enrolled, no further goals		21%	21%	
Not enrolled, has goals for further education		34%	43%	
Enrolled part-time		15%	14%	
Enrolled full-time		29%	22%	
*Household income (as % of federal poverty level)*	2774/2412/2381	159%	216%	
*Health insurance*	2825/2825/2825			810 (29%)
Has any form of health insurance		48%	65%	
No health insurance		52%	35%	
*Employment status*	2774/2780/2735			1259 (46%)
Unemployed		14%	9%	
Employed full-time		45%	58%	
Employed part-time		20%	15%	
Other (homemaker, student, disabled, sick leave, other)		20%	18%	

Note:

^1^ Age at enrollment and race/ethnicity do not vary across waves and are not included in subsequent multivariable fixed-effects models.

### Key measures

We considered a broad range of personal circumstances, specifically relationship status, sexual identity, educational status and aspiration, household income, and employment status as these had been demonstrably linked to pregnancy intentions in previous research.

Because of the influence of relationship status on contraceptive use [13], we distinguish between: married, cohabiting (but not married), actively dating, and those who did not have a current partner or are not actively dating, including those who were divorced or widowed. We also collected information on sexual identity and compared those who are exclusively heterosexual to those who are mostly heterosexual and the sexual minority group, which combines those who are mostly or exclusively heterosexual homosexual/gay or lesbian, bisexual, not sexually attracted to anyone, and those who selected “other.” [26].

For educational status and future goals, we distinguish between those who are not enrolled without future educational goals, those who are not enrolled but who have additional educational aspirations, are enrolled part-time, and are enrolled full-time. We assessed household income relative to the federal poverty level (based on the Centers for Medicaid and Medicare Services (CMS) standard calculation of annual income and household size). We also included an indicator for health insurance status (no health insurance vs. any, including Medicare/Medicaid and private insurance), which can be seen as an indicator of stable health care assess (including access to prenatal care), especially for those with private insurance [27]. Employment status distinguished between being unemployed, working part-time, working full-time, and “other” employment status (which includes self-reported responses of self-employed, homemaker, out of the labor force, disability, among others). We provide a deidentified minimal dataset in the supporting information (S1 File).

### Analytical approach

We first described patterns of change in intended pregnancy timing between enrollment and 12-month follow-up in the analytic sample. In a second step, we used multivariable binary logistic fixed-effects regression analyses to examine the association between personal circumstances and desiring a future pregnancy at follow-up after never desiring a pregnancy at enrollment.

Our fixed-effects models use two data points for each participant (enrollment and 12-month FU). It is important to note that binary logistic fixed-effects regression only include cases where the outcome measure varies between both waves of data, and covariates are only included if they are time-variant; stable individual characteristics such as age at enrollment, race, or ethnicity drop out of fixed-effects models, which, in effect, controls for the effect of time-invariant variables with time-invariant effects. We present adjusted odds ratios for one fully specified multivariable model.

## Results

Table 1 describes the sample characteristics at enrollment and twelve-month follow-up. Table 2 presents data on pregnancy timing intention at baseline and 12-month follow-up. At enrollment, 2805 (99%) participants in the pregnancy intention sample did not intend to get pregnant now or in the next 12 months. We expected this high proportion given that inclusion criteria included a desire to avoid pregnancy in the next 12 months. However, 20 participants indicated that they desired a pregnancy within the next 12 months. Since they stated in the eligibility screening that they wanted to prevent a pregnancy for 12 months, we did not consider this a protocol deviation or reason for exclusion from analysis. Of these 20 participants who represent 0.7% of the sample, 3 (15%) achieved pregnancy at 12-month follow-up (and are shown in Table 3 as desiring a pregnancy “now”). Pregnancy timing intention at enrollment was approximately evenly divided, with one-third of participants stating a desire for pregnancy within 2–5 years, in the next 5–10 years, or never. In the one-year follow-up survey, the proportion of those desiring a pregnancy now or within the next year increased to about 8% (n = 228). Among the 37 participants who reported a “planned” pregnancy between enrollment and 12-month, we lack information about the pregnancy outcome for 3. Among those with information on pregnancy outcome, one participant reported a miscarriage, one participant plans to carry the pregnancy to term and expects to give the baby up for adoption, and 32 plan to carry the pregnancy to term and keep the baby.

**Table 2:** Change in detailed pregnancy timing intentions between enrollment and 12-month follow-up among HER Salt Lake participants.

	Pregnancy Intentions at 12-month follow-up survey					
	Now	Within 12 months	Within 2–5 years	Within 5–10 years	Never	Total
Pregnancy Intentions at Enrollment						
Within 12 months	3	8	8	1	0	20
	(15)	(40)	(40)	(5)	(5)	(100)
Within 2–5 years	56	109	516	83	72	836
	(7)	(13)	(62)	(10)	(9.0)	(100)
Within 5–10 years	12	17	325	551	122	1,027
	(1)	(1)	(32)	(54)	(12)	(100)
Never	8	15	89	96	734	942
	(1)	(2)	(9)	(10)	(78)	(100)
Total	79	149	938	731	928	2,825
	(3)	(5)	(33)	(26)	(33)	(100)

Note: Numbers in parentheses are row percentages. Numbers shaded in grey indicate likely stability in pregnancy intention (n = 2246, 79%). In the box are those whose pregnancy intentions are earlier in the 12-month follow-up compared to the enrollment survey (n = 293, 10%). We observed 113 positive urine pregnancy tests (PUPT) between enrollment at 12-month follow-up. We excluded 76 participants for whom indicated the pregnancy was unintended or we could not ascertain intendedness. Respondents of the 37 PUPT that were the result of trying to become pregnant were classified as desiring a pregnancy “now” at 12-months follow-up survey.

**Table 3:** Binary logistic fixed-effects regression models of changing from never desiring a pregnancy to considering a pregnancy in the future at 12-month follow-up (adjusted odds ratios shown).

	Model 4 aOR (95% Confidence Interval)
*Relationship status*.	
Single (or divorced), not dating	reference
Married	5.35 (0.35–82.76)
Living together with a partner	3.14 (1.30–7.58)
Actively dating	0.68 (0.27–1.72)
*Sexual identity*.	
Exclusively heterosexual	reference
Mostly heterosexual	1.88 (0.46–7.58)
Mostly or exclusively gay/lesbian, bisexual, or other sexuality	1.24 (0.16–9.43)
*Educational enrollment and goals*	
Enrolled full-time	reference
Not enrolled, no further goals	1.95 (0.37–10.17)
Not enrolled, has goals for further education	2.87 (0.75–11.00)
Enrolled part-time	1.97 (0.49–7.90)
*Household income (as % of federal poverty level*, in 10% increments)	1.06 (1.00–1.13)
Currently has *health insurance*	1.17 (0.49–2.83)
*Employment status*.	
Unemployed	reference
Employed full-time	5.94 (1.29–27.36)
Employed part-time	1.20 (0.23–6.23)
Other Employment Status (homemaker, disability, etc.)	3.47 (0.66–18.13)
AIC	143.99
Observations	238
Number of Participants	119

Note: We present a fixed-effects model, which includes only participants for whom outcomes have changed between enrollment and 12-month follow up. 208 participants reported change from never desiring a pregnancy to considering a pregnancy at 12-month follow-up. Analytic sample size is reduced due to missing data on included covariates.

Table 2 presents the shift in pregnancy timing intention data. We found that the majority of the 2,825 participants (2246, 79%) who reported pregnancy intentions at enrollment and 12-month follow-up maintained a consistent pregnancy timing intention over the year, using our broad definition of what considers stability given the measurements categories: We consider participants to have stable intentions if their response categories are the same at 12-month follow up compared to enrollment, or, if their response shifted to the next “earliest” desired pregnancy intention time frame, for example from 5–10 years (at enrollment) to 2–5 years (at follow-up). Someone who desires a pregnancy in 5 years at enrollment (and, due to the passage of time) desires a pregnancy in 4 years at follow-up, will select a different response category. However, our definition of stability might mask that some participants actually change their intentions in ways that are not captured by our response categories. Table 2 illustrates all possible response categories, and the combinations that are considered stable are shaded in grey. At least 579 (20%) reported a change. Among the 942 who reported never intending to become pregnant at enrollment, 208 (22%) changed that intention at 12 months. Over a one-year period, about 10% of participants (n = 293) shifted their pregnancy timing to an earlier time using our narrow definition of what constitutes a change.

We found that multiple aspects of personal circumstances are associated with this shift in pregnancy desires. Switching to cohabitation increases the odds of changing from never desiring a pregnancy to desiring a pregnancy sometime in the future, compared to those who remained single (Table 3). In the fully specified model, changes in sexual identity were not associated with the odds of desiring a pregnancy at 12-month follow-up, but not at enrollment. In supplemental analyses, we examined each of the individual circumstances (education, household income, health care coverage, and employment status) separately to avoid overcontrolling. Results are substantively similar (Table A in S1 Table), but when examined individually, health insurance coverage, rather than household income, is associated with a change in pregnancy intentions.

We examined four dimensions of individual circumstances that have been shown to be linked with pregnancy intentions in previous research. Educational goals and enrollment status, and health insurance coverage were not associated with a switch away from never desiring a pregnancy in a fully specified model. We found that an increase in household income, however, significantly increased the change to considering a pregnancy after reporting never wanting a pregnancy at enrollment (aOR 1.06, 95% CI 1.00–1.34). Lastly, a change from unemployment to full-time employment also increased the odds of desiring a pregnancy at follow-up for those who never wanted a pregnancy at enrollment (aOR 5.94, 95% CI 1.29–27.36). In supplemental analyses that included education, household income, health care coverage, and employment status separately, we show a link between health insurance coverage and change in pregnancy intentions, but not for household income (Table A in S1 Table).

Additional supplemental analyses (Table B in S1 Table) examined the association among personal circumstances with desiring a pregnancy now or in the next 12 months and found trends similar to those who switched from never desiring pregnancy to considering one in the future.

## Discussion

Our study shows that even over the relatively short period of 12 months, there are substantial changes in pregnancy intentions. We find that just under 20% of participants changed their pregnancy intentions, which might be an undercount given our broad definition of who we count as having stable intentions. Additionally, about 22% of those who initially indicated they never wanted a pregnancy changed their mind over the course of 12 months. Our findings underline the fluidity of pregnancy intentions and attitudes towards pregnancy, even among those who state they never wanted a pregnancy. This study highlights the need to more frequently assess whether someone’s current contraceptive strategy is helping them meet their reproductive goals [5,12,28,29]. We find that markers of stability, such as getting entering married and cohabitation, increased household income, gaining access to health insurance, or switching from unemployment to full-time work (and also entering “other” employment status), are associated with a change from never desiring a pregnancy to desiring one now or in the future over a mere 12-month period. This fits with existing research that indicates that individuals seek to achieve economic stability before planning a pregnancy [16,28,29].

The strengths of this study include a large cohort with high follow-up rates and detailed information about participants’ personal circumstances. Our sample includes a group of participants who is quite economically disadvantaged at time of enrollment, with high levels of poverty and lack insurance coverage. Likely because of this, we see much change even over the short period of one year. The limitations include the predetermined time ranges in our pregnancy intention question and lack of an “uncertain” option at all timepoints. Given the broad categories, we used a very conservative approach to categorize someone as changing intentions. As a result, some who we consider having “stable” intentions may actually have changed their pregnancy intention timing. It is also possible that participants underreported their pregnancy desires at enrollment to qualify for study participation and free contraception. The questionnaire design did not fully take into account the experiences of participants who were pregnant, and the study only included participants who, at screening for participation, desired to avoid pregnancy for at least 12 months. Our study cannot assess the association between pregnancies and pregnancy intentions for subsequent, higher order pregnancies. We excluded individuals who reported unplanned pregnancies or pregnancies with missing information on whether they were planned. Our question about pregnancy intentions may have been confusing for people with current or recent pregnancies. Future research will more comprehensively address the experience and trajectories of participants’ pregnancies, and their impact on future pregnancy intentions. Moreover, our study does not address the complicated issues of how we talk (and think) about pregnancy intentions in terms of desires or plans. In this study we treat plans, intentions, and desires interchangeable terms. Future research might want to consider survey experiments to see how sensitive response are to the language used in questions. Further, our measure of employment is not very nuanced and, while we take into account whether or not participants have additional educational goals, future research needs to examine the association between completing a degree or certificate and pregnancy intentions. Future research also needs to examine the trajectories of those who were uncertain about their pregnancy intentions at enrollment, as they may be particularly likely to change their intentions. Our use of fixed-effects models relies on the assumption that the effect of time invariant characteristics (i.e., race) is stable over time, an assumption, which future research might want to explore in more detail.

Nevertheless, the implications of our findings are manifold. Our findings are important context for providers, as research on contraceptive switching and continuation has shown that providers might be reluctant to remove LARC after short usage time [30]. While providers might already anticipate a change in pregnancy intentions due to marriage or cohabitation with a partner, our study highlights that a more in-depth understanding of clients’ personal circumstances matters. Providers need to anticipate that contraceptive needs change rapidly and should embrace and support changing desires and clients’ needs.

These findings support that individuals’ reproductive healthcare needs (including contraception) are dynamic and suggest that they may be linked to situational changes in people’s lives. This study’s focus on those who have concrete intention is likely to result in an underestimation of the extent of individual change in pregnancy intention over time. Our findings reinforce the importance of regularly asking clients about their desired reproductive goals, ideal timing for parenthood, as well as pregnancy timing, even after they have recently committed to long-acting methods or stated they never desire a pregnancy. This could de-stigmatize contraceptive switching and discontinuation and encourage providers to be responsive to client needs.

## Supporting information

S1 Codebook (PDF)

S1 File Deidentified minimal data set. (DTA)

S1 Table Supplemental tables A, B, and C. (DOCX)
