# Supplementary material for: Changing lives, dynamic plans: Prospective assessment of 12-month changes in pregnancy timing intentions and personal circumstances using data from HER Salt Lake
Source: PLoS One. 2021 Sep 20;16(9):e0257411. doi: 10.1371/journal.pone.0257411 (PMC8451991; doi:10.1371/journal.pone.0257411)
Supplement: S1 Codebook — (PDF) [file pone.0257411.s001.pdf]

Project Home | Project Setup | Online Designer | Data Dictionary | Codebook

Data Dictionary Codebook

08/08/2021 12:31pm

Collapse all instruments

#	Variable / Field Name	Field Label *Field Note*	Field Attributes (Field Type, Validation, Choices, Calculations, etc.)
Instrument: **Clinic Intake Form** (clinic_intake_form) Enabled as survey			Expand
Instrument: **Phase/Method Switch** (phasemethod_switch) Enabled as survey			Expand
Instrument: **Final Study Status** (schedule)			Expand
Instrument: **Enrollment Survey** (enrollment_survey) Enabled as survey			Collapse
121	reason_coming	Section Header: *We want to ask you about your visit to the clinic today.*<br>What is the main reasons you came to the clinic today?<br>*select all that apply*	checkbox, Required<br>1 reason_coming___1 Annual check-up and/or well woman exam, including pap smear<br>2 reason_coming___2 To get birth control or change methods<br>3 reason_coming___3 To get emergency contraception (the morning after pill)<br>4 reason_coming___4 Pregnancy testing<br>5 reason_coming___5 STI/STD testing<br>6 reason_coming___6 Abortion services<br>7 reason_coming___7 To get free or low cost birth control<br>8 reason_coming___8 To participate in this study<br>99 reason_coming___99 I prefer not to answer<br>88 reason_coming___88 Other
122	reason_other<br>Show the field ONLY if:<br>[reason_coming(88)] = '1'	If "other," please describe your reason for coming to the clinic today.	notes
123	heard_of_services	How did you hear about the services that this clinic provides?<br>*select all that apply*	checkbox, Required<br>1 heard_of_services___1 Family or friend<br>2 heard_of_services___2 Partner, Boyfriend/Girlfriend, Spouse<br>3 heard_of_services___3 School class or school teacher<br>4 heard_of_services___4 Health care provider or other clinic<br>5 heard_of_services___5 Facebook/Twitter or other social media<br>12 heard_of_services___12 Planned Parenthood advertising (ProUtah, etc.)

			6 | heard_of_services___6 | The website www.Bedsider.org<br>7 | heard_of_services___7 | Other website<br>8 | heard_of_services___8 | Television, radio, or newspaper<br>10 | heard_of_services___10 | I have been to the clinic before<br>11 | heard_of_services___11 | Text message sent directly to me<br>13 | heard_of_services___13 | www.hersaltlake.org<br>9 | heard_of_services___9 | Other<br>88 | heard_of_services___88 | I don't know<br>99 | heard_of_services___99 | I prefer not answer
124	heard_other<br>Show the field ONLY if:<br>[heard_of_services(7)] = '1' or [heard_of_services(9)] = '1'	If "other," please describe how you heard about the services we provide.	text
125	visited_hersl	Have you ever visited the website www.hersaltlake.org?	radio<br>0 | No<br>1 | Yes<br>88 | I don't know<br>99 | I prefer not to answer
126	hersl_heard<br>Show the field ONLY if:<br>[heard_of_services(12)] = '1' or [visited_proutah] = '1'	Where did you learn about www.hersaltlake.org?	checkbox<br>1 | hersl_heard___1 | Word of mouth (family or friend)<br>2 | hersl_heard___2 | Online Ad Banner<br>3 | hersl_heard___3 | Facebook<br>4 | hersl_heard___4 | Instagram<br>5 | hersl_heard___5 | Twitter<br>6 | hersl_heard___6 | Newspaper, Radio,<br>9 | hersl_heard___9 | Other
127	visited_proutah	Have you ever visited the website www.proutah.org?	radio<br>0 | No<br>1 | Yes<br>88 | I don't know<br>99 | I prefer not to answer
128	proutah_heard<br>Show the field ONLY if:<br>[heard_of_services(12)] = '1' or [visited_proutah] = '1'	Where did you see Planned Parenthood advertising or learn about proutah.org?	checkbox<br>1 | proutah_heard___1 | Online publications<br>2 | proutah_heard___2 | Public transportation<br>3 | proutah_heard___3 | Billboards<br>4 | proutah_heard___4 | Radio<br>5 | proutah_heard___5 | Social Media<br>9 | proutah_heard___9 | Other
129	visited_bedsider	Have you ever visited the website www.bedsider.org?	radio<br>0 | No<br>1 | Yes

			88 I don't know<br>99 I prefer not to answer
130	learned_of_besider<br><br>Show the field ONLY if:<br>[visited_bedsider] = '1'	If you have visited the website www.bedsider.org, how did you learn about it?<br>*select all that apply*	checkbox<br>1 learned_of_besider___1 Family or friend<br>2 learned_of_besider___2 Partner, Boyfriend/Girlfriend, Spouse<br>3 learned_of_besider___3 School class or school teacher<br>4 learned_of_besider___4 Health care provider or other clinic<br>5 learned_of_besider___5 Facebook/Twitter or other social media<br>6 learned_of_besider___6 The website www.Bedsider.org<br>7 learned_of_besider___7 Other website<br>11 learned_of_besider___11 Message sent directly to my phone<br>8 learned_of_besider___8 Television, radio, or newspaper<br>9 learned_of_besider___9 Other<br>10 learned_of_besider___10 I have been to the clinic before<br>88 learned_of_besider___88 I don't know<br>99 learned_of_besider___99 I prefer not answer
131	other_learned<br><br>Show the field ONLY if:<br>[learned_of_besider(8)] = '1' or [learned_of_besider(6)] = '1'	If other, please specify:	text
132	usborn	Section Header: *Participant Background First, please tell us a little bit more about yourself. Your answers will NOT impact participation in the study, clinical care, or any services you receive.*<br><br>Were you born in the United States?	radio, Required<br>0 No<br>1 Yes<br>99 I prefer not to answer
133	birth_country<br><br>Show the field ONLY if:<br>[usborn] = '0'	In what country were you born?	text
134	usarrival_yr<br><br>Show the field ONLY if:<br>[usborn] = '0'	What year did you move to the United States?	text (number)
135	gender_id	We understand that not everyone who needs or wants birth control identifies as a woman.<br><br>Please select the word(s) you use to describe yourself, or select self-describe and you can write in the word(s) you use.<br>*check all that apply*	checkbox<br>1 gender_id___1 Woman<br>2 gender_id___2 Man<br>3 gender_id___3 Non-binary<br>4 gender_id___4 Transgender<br>5 gender_id___5 Prefer to self-describe
136	gender_txt	Please describe yourself.	text
137	race	Which of the following best describes your ethnicity/race?	checkbox, Required

			1 race___1 White<br>2 race___2 Hispanic or Latina<br>3 race___3 Asian<br>4 race___4 Native Hawaiian or Pacific Islander<br>5 race___5 American Indian or Alaska Native<br>6 race___6 African American or Black<br>7 race___7 Other<br>88 race___88 I don't know<br>99 race___99 I prefer not to answer
138	race_other<br>Show the field ONLY if:<br>[race(7)] = '1'	If other please describe.	text
139	religion	Which of the following best describes your religious identity?	radio<br>0 Not Religious<br>1 Christian (Protestant, Evangelical, Mainline, etc)<br>2 Catholic<br>3 Mormon<br>4 Jewish<br>5 Muslim<br>6 Other<br>88 Don't know or prefer not to answer
140	religion_txt<br>Show the field ONLY if:<br>[religion] = '6'	Please describe your religious identity.	text
141	relationship_status	Which of the following best describes your current relationship situation?	radio, Required<br>1 Married<br>2 Not married, but living together or in a committed relationship<br>3 Actively dating, but NOT in a committed relationship<br>4 Divorced/Separated<br>5 Single, not in a relationship<br>6 Widowed<br>7 Other<br>99 I prefer not to answer
142	relationship_oth<br>Show the field ONLY if:<br>[relationship_status] = '7'	Other, please describe	text
143	sexual_identitiy	Please choose the description that best fits how you think of yourself.	radio, Required<br>1 Exclusively Heterosexual (Straight)<br>5 Mostly Heterosexual<br>3 Bisexual (Attracted to both Men and Women)<br>6 Mostly Gay/Lesbian<br>2 Exclusively Homosexual (Gay/Lesbian)<br>7 Not sexually attracted to either males or

			females<br>4 Other<br>88 I don't know<br>99 I prefer not to answer<br><br>Field Annotation: The categories of mostly heterosexual and mostly gay as well as not sexually attracted to either were added 2/25/16 per Bethany Everett recomendations
144	sexwith	In the last 12 months have you had sex with...	radio<br>0 Not currently sexually active<br>1 Males only<br>2 Both males and females<br>3 Females only<br>88 I prefer not to answer
145	lifetime_female_partners	Considering all types of sexual activity, how many female partners have you EVER had sex with? (If you can't remember exactly, just make your best guess.)	text (number), Required
146	last_year_female_partners<br>Show the field ONLY if:<br>[lifetime_female_partners] > 0	Considering all types of sexual activity, with how many female partners have you had sex in the past 12 months, even if only one time?	text (number), Required
147	insurance	What type of medical insurance do you currently have?	radio, Required<br>1 None<br>2 Medicaid<br>4 Insurance through your job or that you buy on your own<br>6 Student health insurance<br>7 Parent's insurance<br>5 Military or VA (Champus, ChampVA, Tricare)<br>3 Disability or Medicare<br>88 I don't know<br>99 I prefer not to answer
148	employment	Section Header: *The next few questions are about your current employment.*<br>What best describes your current employment status?<br>*select all that apply*	checkbox, Required<br>0 employment___0 Unemployed<br>1 employment___1 Working full-time (at least 30 hrs/wk)<br>2 employment___2 Working part-time (less than 30 hrs/wk)<br>3 employment___3 Disabled, sick leave, family leave<br>4 employment___4 Retired<br>5 employment___5 Homemaker<br>6 employment___6 Student<br>8 employment___8 Other<br>99 employment___99 I prefer not to answer
149	employment_oth<br>Show the field ONLY if:<br>[employment(8)] = '1'	If other employment, please describe.	text

150	employ_hours<br><br>Show the field ONLY if:<br>[employment(1)] = '1' or [employment(2)] = '1'	How many hours do you work each week?<br>*Please guess if you are not sure*	text (number)
151	hourly_wage<br><br>Show the field ONLY if:<br>[employment(2)] = '1' or [employment(1)] = '1'	What is your hourly wage?<br>*Example 7.25 if you make $7.25 per hour....Please estimate if you are not sure*	text
152	employ_type<br><br>Show the field ONLY if:<br>[employment(1)] = '1' or [employment(2)] = '1'	What kind of work do you do?	notes
153	lookingforwork	Are you currently looking for work, additional work, or different work?	radio<br>0 No<br>1 Yes<br>88 Don't know<br>99 I prefer not to answer this question
154	education	Section Header: *The next few questions are about your education and education level of your parents.*<br><br>What best describes the highest level of education you have COMPLETED SO FAR?	radio, Required<br>1 11th grade or less<br>2 12th grade (completed high school or GED)<br>3 Vocational/technical training<br>4 Associate degree or some college<br>5 4-year college degree (BA/BS)<br>6 Any graduate or professional education (any time in a Masters, JD, PhD, MD, etc. program)<br>88 I don't know<br>99 I prefer not to answer
155	inschool	Are you currently in school, either full-time or part-time?	radio<br>0 I'm not currently in school<br>1 Part-time<br>2 Full-time<br>99 I prefer not to answer this question
156	education_plans	What best describes your PLANS for the highest level of education in the future?	radio, Required<br>0 None, I am done with school and do not have plans to go back<br>1 I plan to finish high school or GED<br>2 Get vocational/technical training<br>3 Get an associate degree<br>4 Get a 4-year college degree (BA/BS)<br>5 Get graduate or professional education (Masters, JD PhD, MD)<br>88 I don't know<br>99 I prefer not to answer
157	student_grddate<br><br>Show the field ONLY if:<br>[education_plans] = '1' or [education_plans] = '2' or [educat	What date do you expect to graduate?	text (date_mdy)

	ion_plans] = '3' or [education_plans] = '4' or [education_plans] = '5'		
158	education_mom	What is the highest level of education your mother (or female guardian) completed?	radio, Required<br>1 Less than High School<br>2 High School degree or GED<br>3 Vocational/technical training<br>4 Associate degree or some college<br>5 4-year college degree (BA/BS)<br>6 Any graduate or professional education (any time in a Masters, JD, PhD, MD, etc.)<br>88 I don't know<br>99 I prefer not to answer
159	education_dad	What is the highest level of education your father (or male guardian) completed?	radio, Required<br>1 Less than High School<br>2 High School degree or GED<br>3 Vocational/technical training<br>4 Associate degree or some college<br>5 4-year college degree (BA/BS)<br>6 Any graduate or professional education (any time in a Masters, JD, PhD, MD, etc.<br>88 I don't know<br>99 I prefer not to answer
160	income_annual	Section Header: *Thank you for you answers. Just a few more questions about your financial situation. Again, these will not impact your participation or clinical care.*<br>Please select the range of your yearly household income?	radio, Required<br>1 Less than $10,000<br>2 $10,000-$19,999<br>3 $20,000-$29,999<br>4 $30,000-$39,999<br>5 $40,000-$49,999<br>6 $50,000-$59,999<br>7 $60,000-$69,999<br>8 $70,000-$79,999<br>9 $80,000 or more<br>88 I don't know<br>99 I prefer not to answer this question
161	income_annual_exact	What is your best estimate of your yearly household income?<br>*8,500 if your income is $8,500 per year*	text, Required
162	dependents	How many people are in your household?<br>*including you*	dropdown, Required<br>1 1<br>2 2<br>3 3<br>4 4<br>5 5<br>6 6<br>7 7

			8 8<br>9 9<br>10 10<br>11 11<br>12 12<br>13 13<br>14 14<br>15 15+
163	children<br><br>Show the field ONLY if:<br>[dependents] > 1	How many children under the age of 18 do you have living with you?	text
164	incomesource	Please check all of the following that have been a source of income in the last month:<br>*select all that apply*	checkbox<br>1 incomesource___1 Self<br>2 incomesource___2 Spouse or Partner<br>3 incomesource___3 Other family member<br>4 incomesource___4 Government assistance<br>5 incomesource___5 Other<br>99 incomesource___99 I prefer not to answer
165	oth_income_txt<br><br>Show the field ONLY if:<br>[incomesource(5)] = '1'	What other sources of income did you receive in the past month?	text
166	income_mo<br><br>Show the field ONLY if:<br>[incomesource(1)] = '1'	How much money did you make last month?	radio, Required<br>1 None<br>2 $1-$400<br>3 $401-$800<br>4 $801-$1,200<br>5 $1,201-$1,600<br>6 $1,601-$2,000<br>7 $2,001-$2,400<br>8 $2,401-$2,800<br>9 More than $2,800<br>88 Don't know<br>99 I prefer not to answer
167	pt_income_mo<br><br>Show the field ONLY if:<br>[incomesource(2)] = '1'	How much money did your partner or spouse make last month?	radio<br>1 None<br>2 $1-$400<br>3 $401-$800<br>4 $801-$1,200<br>5 $1,201-$1,600<br>6 $1,601-$2,000<br>7 $2,001-$2,400<br>8 $2,401-$2,800<br>9 More than $2,800<br>88 Don't know

			99 I prefer not to answer
168	fam_income_mo<br>Show the field ONLY if:<br>[incomesource(3)] = '1'	How much money did your other family members contribute to your household income?	radio<br>1 None<br>2 $1-$400<br>3 $401-$800<br>4 $801-$1,200<br>5 $1,201-$1,600<br>6 $1,601-$2,000<br>7 $2,001-$2,400<br>8 $2,401-$2,800<br>9 More than $2,800<br>88 Don't know<br>99 I prefer not to answer
169	gov_income_mo<br>Show the field ONLY if:<br>[incomesource(4)] = '1'	How much money did government assistance contribute to your household income last month?	radio<br>1 None<br>2 $1-$400<br>3 $401-$800<br>4 $801-$1,200<br>5 $1,201-$1,600<br>6 $1,601-$2,000<br>7 $2,001-$2,400<br>8 $2,401-$2,800<br>9 More than $2,800<br>88 Don't know<br>99 I prefer not to answer
170	oth_income_mo<br>Show the field ONLY if:<br>[incomesource(5)] = '1'	How much money did you receive from other sources last month?<br>*this includes child support*	radio<br>1 None<br>2 $1-$400<br>3 $401-$800<br>4 $801-$1,200<br>5 $1,201-$1,600<br>6 $1,601-$2,000<br>7 $2,001-$2,400<br>8 $2,401-$2,800<br>9 More than $2,800<br>88 Don't know<br>99 I prefer not to answer
171	childsupport_planned<br>Show the field ONLY if:<br>[children] >= 1	Were you supposed to receive any child support in the last 4 weeks?	radio<br>0 No<br>1 Yes<br>99 I prefer not to answer
172	childsupport_amt<br>Show the field ONLY if:<br>[childsupport_actual] = '1'	How much child support were you supposed to receive?	text

173	childsupport_actual<br>Show the field ONLY if:<br>[children] >= 1 or [childsupport_planned] ='1'	Did you receive any child support in the last 4 weeks?	radio<br>0 No<br>1 Yes<br>99 I prefer not to answer this question
174	childsupport_amtactual<br>Show the field ONLY if:<br>[childsupport_actual] = '1'	How much child support did you receive in the last 4 weeks?	text
175	typehome	Which of the following best describes your current housing situation?	radio<br>0 Homeless<br>1 Shelter<br>2 Mobile home<br>3 Apartment<br>4 Single-family house<br>6 Other<br>99 I prefer not to answer this question
176	otherhousing<br>Show the field ONLY if:<br>[typehome] = '6'	Describe type of housing	text
177	asst_food	Section Header: *We will now ask a few questions about public assistance that you may receive: Do you currently receive:*<br>Food stamps	radio (Matrix), Required<br>0 No<br>1 Yes<br>99 Prefer not to answer
178	asst_wic	WIC (Women, Infants and Children)	radio (Matrix), Required<br>0 No<br>1 Yes<br>99 Prefer not to answer
179	asst_welfare	Welfare	radio (Matrix), Required<br>0 No<br>1 Yes<br>99 Prefer not to answer
180	asst_unemployment	Unemployment Benefits	radio (Matrix), Required<br>0 No<br>1 Yes<br>99 Prefer not to answer
181	pay_trans	Section Header: *Just a few more questions about your economic situation. During the past 12 months, have you had trouble paying for the following:*<br>Transportation	radio (Matrix), Required<br>0 No<br>1 Yes<br>99 Prefer not to answer
182	pay_housing	Housing	radio (Matrix), Required<br>0 No<br>1 Yes<br>99 Prefer not to answer
183	pay_health	Medical care or medications	radio (Matrix), Required

			0 No<br>1 Yes<br>99 Prefer not to answer
184	pay_food	Food	radio (Matrix), Required<br>0 No<br>1 Yes<br>99 Prefer not to answer
185	enoughmoney	During the past month, how often would you say you had enough money to meet your basic living needs such as food, housing and transportation?	radio<br>1 All the time<br>2 Most of the time<br>3 Some of the time<br>4 Rarely<br>5 Never<br>88 Don't know<br>99 I prefer not to answer
186	hx_pregnancy	Section Header: *Now we want to ask you some questions about your pregnancy history and outcomes.*<br>Have you ever been pregnant before?	radio, Required<br>0 No<br>1 Yes<br>99 Prefer not to answer
187	life_preg<br>Show the field ONLY if:<br>[hx_pregnancy] = '1'	How many times in your life have you been pregnant	dropdown<br>0 0<br>1 1<br>2 2<br>3 3<br>4 4<br>5 5<br>6 6<br>7 7<br>8 8<br>9 9<br>10 10+
188	life_lb<br>Show the field ONLY if:<br>[hx_pregnancy] = '1'	How many times in your life have you had a live birth?	dropdown<br>0 0<br>1 1<br>2 2<br>3 3<br>4 4<br>5 5<br>6 6<br>7 7<br>8 8<br>9 9<br>10 10+

189	life_miss<br><br>Show the field ONLY if:<br>[hx_pregnancy] = '1'	How many times in your life have you had a miscarriage?	dropdown<br>0 0<br>1 1<br>2 2<br>3 3<br>4 4<br>5 5<br>6 6<br>7 7<br>8 8<br>9 9<br>10 10+
190	life_abortion<br><br>Show the field ONLY if:<br>[hx_pregnancy] = '1'	How many times in your life have you had an abortion?	dropdown<br>0 0<br>1 1<br>2 2<br>3 3<br>4 4<br>5 5<br>6 6<br>7 7<br>8 8<br>9 9<br>10 10+
191	life_still<br><br>Show the field ONLY if:<br>[hx_pregnancy] = '1'	How many times in your life have you had a stillbirth?	dropdown<br>0 0<br>1 1<br>2 2<br>3 3<br>4 4<br>5 5<br>6 6<br>7 7<br>8 8<br>9 9<br>10 10+
192	life_tubal<br><br>Show the field ONLY if:<br>[hx_pregnancy] = '1'	How many times in your life have you had an ectopic (tubal) pregnancy?	dropdown<br>0 0<br>1 1<br>2 2<br>3 3<br>4 4<br>5 5

			6 6<br>7 7<br>8 8<br>9 9<br>10 10+
193	life_adopt<br><br>Show the field ONLY if:<br>[life_lb] = '1' or [life_lb] = '2' or [life_lb] = '3' or [life_lb] = '4' or [life_lb] = '5' or [life_lb] = '6' or [life_lb] = '7' or [life_lb] = '8' or [life_lb] = '9' or [life_lb] = '10'	How many times in your life have you placed a child for adoption?	dropdown<br>0 0<br>1 1<br>2 2<br>3 3<br>4 4<br>5 5<br>6 6<br>7 7<br>8 8<br>9 9<br>10 10+
194	life_unplanned<br><br>Show the field ONLY if:<br>[hx_pregnancy] = '1'	How many times in your life have you been pregnant when you did not want to be?	dropdown<br>0 0<br>1 1<br>2 2<br>3 3<br>4 4<br>5 5<br>6 6<br>7 7<br>8 8<br>9 9<br>10 10+
195	age_firstpreg<br><br>Show the field ONLY if:<br>[hx_pregnancy] = '1'	How old were you when you became pregnant for the FIRST time?	text (number)
196	date_end_last_pregnancy<br><br>Show the field ONLY if:<br>[hx_pregnancy] = '1'	When did your MOST RECENT pregnancy end? If you can't remember the exact date, please make your best guess.	text (date_mdy)
197	preg_outcome<br><br>Show the field ONLY if:<br>[hx_pregnancy] = '1'	How did your most recent pregnancy end?	radio<br>1 Miscarriage<br>2 Abortion<br>3 Preterm live birth (less than 37 weeks)<br>4 Term live birth (37 weeks or more)<br>5 Ectopic (tubal)<br>6 Stillbirth (20 weeks or more)<br>99 Prefer not to answer
		Section Header: *Now we would like to ask you about your plans for future*	

198	future_children	*children, ideal timing, and feelings surrounding pregnancy.*<br>Do you think you would like to have children (or more children) in the future?	radio<br>0 No<br>1 Yes<br>99 I don't know
199	preg_plans	What are your future pregnancy plans?	radio<br>0 I do not plan on getting pregnant at any time in the future.<br>1 I would like to get pregnant in the next year.<br>2 I would like to get pregnant in the next 2-5 years (but not in the next year).<br>3 I would like to get pregnant in the next 5-10 years but not before then.<br>4 I am uncertain of if or when I would like to become pregnant.<br>99 Other
200	preg_plans_oth<br>Show the field ONLY if:<br>[preg_plans] = '99'	What are your pregnancy plans?	text
201	preg_intention_importance<br>Show the field ONLY if:<br>[future_children] = '1' or [future_children] = '99'	How important is it to you to not get pregnant until you are ready?	slider<br>Slider labels: not at all important, , the most important<br>Custom alignment: RH
202	preg_avoid_import<br>Show the field ONLY if:<br>[future_children] = '0'	How important is it to you to not get pregnant now or in the future?	slider<br>Slider labels: not at all important, , the most important<br>Custom alignment: RH
203	preg_happy	How would you feel about getting pregnant in the next month?	slider<br>Slider labels: worst feeling you can imagine, , happiest you could possibly feel<br>Custom alignment: RH
204	preg_impact	Please tell us a bit more about how a pregnancy now or in the next few weeks would affect your life.	notes
205	bc_hx	Section Header: *Now we want to ask you some questions about methods you have used to prevent pregnancy.*<br>What method(s) to prevent pregnancy have you EVER used in the past?<br>*select all that apply*	checkbox<br>1 bc_hx___1 Contraceptive Implant (Nexplanon)<br>2 bc_hx___2 Copper IUD (Paragard)<br>3 bc_hx___3 Hormonal IUD (Mirena)<br>4 bc_hx___4 Hormonal IUD (Liletta)<br>5 bc_hx___5 Other hormonal IUD (Skyla)<br>6 bc_hx___6 Injection (Depo-Provera)<br>7 bc_hx___7 Combined oral contraceptive pill (The Pill)<br>8 bc_hx___8 Progestin Only Pill (Minipill)<br>9 bc_hx___9 Contraceptive patch (Xulane or OrthoEvra)<br>10 bc_hx___10 Vaginal ring (NuvaRing)<br>11 bc_hx___11 Male condom<br>12 bc_hx___12 Female condom<br>13 bc_hx___13 Cervical cap or sponge

			14 bc_hx___14 Spermicide<br>15 bc_hx___15 Diaphragm<br>16 bc_hx___16 Fertility Awareness Method / Natural Family Planning / Rhythm Method<br>17 bc_hx___17 Withdrawal<br>18 bc_hx___18 Levonorgestrel Emergency Contraception (Plan B/Next Choice)<br>19 bc_hx___19 Ulipristal EC Pill (Ella)<br>99 bc_hx___99 Other<br>0 bc_hx___0 None; I've never used contraception
206	bc_hx_other<br>Show the field ONLY if:<br>[bc_hx(99)] = '1'	If other, please specify:	text
207	bc_recent	What method(s) have you used in the last 4 weeks?<br><br>This should not include the method you are receiving today.<br>*select all that apply*	checkbox<br>1 bc_recent___1 Contraceptive Implant (Nexplanon)<br>2 bc_recent___2 Copper IUD (Paragard)<br>3 bc_recent___3 Hormonal IUD (Mirena)<br>4 bc_recent___4 Hormonal IUD (Liletta)<br>5 bc_recent___5 Other hormonal IUD (Skyla)<br>6 bc_recent___6 Injection (Depo-Provera)<br>7 bc_recent___7 Combined oral contraceptive pill (The Pill)<br>8 bc_recent___8 Progestin Only Pill (Minipill)<br>9 bc_recent___9 Contraceptive patch (Xulane or OrthoEvra)<br>10 bc_recent___10 Vaginal ring (NuvaRing)<br>11 bc_recent___11 Male condom<br>12 bc_recent___12 Female condom<br>13 bc_recent___13 Cervical cap or sponge<br>14 bc_recent___14 Spermicide<br>15 bc_recent___15 Diaphragm<br>16 bc_recent___16 Fertility Awareness Method / Natural Family Planning / Rhythm Method<br>17 bc_recent___17 Withdrawal<br>18 bc_recent___18 Levonorgestrel Emergency Contraception (Plan B/Next Choice)<br>19 bc_recent___19 Ulipristal EC Pill (Ella)<br>20 bc_recent___20 Abstinence<br>99 bc_recent___99 Other<br>0 bc_recent___0 None; I have not used anything to avoid pregnancy in the last 4 weeks

208	bc_recent_oth<br>Show the field ONLY if:<br>[bc_recent(99)] = '1'	If other, please specify:	text
209	bc_primary_duration<br>Show the field ONLY if:<br>[bc_recent(1)] = '1' or [bc_recent(2)] = '1' or [bc_recent(3)] = '1' or [bc_recent(4)] = '1' or [bc_recent(5)] = '1' or [bc_recent(6)] = '1' or [bc_recent(7)] = '1' or [bc_recent(8)] = '1' or [bc_recent(9)] = '1' or [bc_recent(10)] = '1' or [bc_recent(11)] = '1' or [bc_recent(12)] = '1' or [bc_recent(13)] = '1' or [bc_recent(14)] = '1' or [bc_recent(15)] = '1' or [bc_recent(16)] = '1' or [bc_recent(99)] = '1'	How long have you been on this method of birth control?<br><br>If you are using multiple methods answer for any hormonal method.	radio, Required<br>1 less than 3 months<br>2 3 to 6 months<br>3 6 months to 1 year<br>4 1 to 2 years<br>5 2 to 3 years<br>6 more than 3 years<br>88 I don't know<br>99 I prefer not to answer
210	bc_primary_bc_more<br>Show the field ONLY if:<br>[bc_primary_duration] = '6'	How many years have you used this method of birth control?	text
211	satisfaction_bc	Overall, how satisfied are you with the method you were using during the previous 4 weeks?	radio, Required<br>1 Completely satisfied<br>2 Somewhat satisfied<br>3 Neither satisfied or dissatisfied<br>4 Somewhat dissatisfied<br>5 Completely dissatisfied<br>99 I prefer not to answer
212	confidence_bc	Overall, how confident were you that the method you have been using the past 4 weeks will prevent pregnancy?	radio, Required<br>5 Very high confidence<br>4 High confidence<br>3 Moderate confidence<br>2 Low confidence<br>1 Very low or no confidence<br>99 I prefer not to answer
213	control_over_pregnancy	Please rate your agreement or disagreement with the following statement: "I feel that I have control over whether or not I get pregnant."	radio, Required<br>1 I strongly agree<br>2 I somewhat agree<br>3 I neither agree nor disagree<br>4 I somewhat disagree<br>5 I strongly disagree<br>99 I prefer not to answer
214	heard_iud	Before today had you heard of the IUD?	yesno<br>1 Yes<br>0 No
215	interest_iud	Which best describes your level of interest in getting an IUD for birth control today?	radio<br>0 Not at all interested<br>1 Somewhat interested

			2 Extremely interested
216	free_iud	If you could have an IUD placed today for free, would you want one?	radio<br>0 No<br>1 Yes<br>99 Not sure
217	cantget_iud	Did you want an IUD but were not able to get it today?	radio<br>0 No<br>1 Yes<br>99 Not sure
218	cantget_iud_why<br>Show the field ONLY if:<br>[cantget_iud]=1	If yes, explain why:	text
219	heard_implant	Before today had you heard of the contraceptive implant, Nexplanon?	yesno<br>1 Yes<br>0 No
220	interest_implant	Which best describes your level of interest in getting an implant for birth control today?	radio<br>0 Not at all interested<br>1 Somewhat interested<br>2 Extremely interested
221	free_implant	If you could have an implant placed today for free, would you want one?	radio<br>0 No<br>1 Yes<br>99 Not sure
222	cantget_implant	Did you want an implant but were not able to get it today?	radio<br>0 No<br>1 Yes<br>99 Not sure
223	cantget_implant_why<br>Show the field ONLY if:<br>[cantget_implant]=1	If yes, explain why:	text
224	reason_hormones	Section Header: *How important are each of the following characteristics to you in deciding which birth control method to use?*<br>It doesn't contain hormones	radio (Matrix)<br>1 Not at all important<br>2 Slightly important<br>3 Quite important<br>4 Extremely important<br>99 I don't know/ I prefer not to answer
225	reason_partner	It is acceptable to my partner	radio (Matrix)<br>1 Not at all important<br>2 Slightly important<br>3 Quite important<br>4 Extremely important<br>99 I don't know/ I prefer not to answer
226	reason_interrupt	It doesn't interrupt sex	radio (Matrix)<br>1 Not at all important

			2 Slightly important<br>3 Quite important<br>4 Extremely important<br>99 I don't know/ I prefer not to answer
227	reason_libido	It doesn't reduce my libido	radio (Matrix)<br>1 Not at all important<br>2 Slightly important<br>3 Quite important<br>4 Extremely important<br>99 I don't know/ I prefer not to answer
228	reason_religion	It is in line with my religious beliefs	radio (Matrix)<br>1 Not at all important<br>2 Slightly important<br>3 Quite important<br>4 Extremely important<br>99 I don't know/ I prefer not to answer
229	reason_friends	It is recommended by my friend(s)	radio (Matrix)<br>1 Not at all important<br>2 Slightly important<br>3 Quite important<br>4 Extremely important<br>99 I don't know/ I prefer not to answer
230	reason_effective	It is the most effective method	radio (Matrix)<br>1 Not at all important<br>2 Slightly important<br>3 Quite important<br>4 Extremely important<br>99 I don't know/ I prefer not to answer
231	reason_se	It doesn't have side effects	radio (Matrix)<br>1 Not at all important<br>2 Slightly important<br>3 Quite important<br>4 Extremely important<br>99 I don't know/ I prefer not to answer
232	reason_safe	It is safe for me to use	radio (Matrix)<br>1 Not at all important<br>2 Slightly important<br>3 Quite important<br>4 Extremely important<br>99 I don't know/ I prefer not to answer
233	gen_last4wk_a	Section Header: *Now we want to ask you a few questions about certain aspects of your health and well-being that may be related to your menstrual cycle. You may still experience these things if you don't have a period. In the past 4 weeks, have any the following health issues been a problem for you?*<br>Headaches	radio (Matrix)<br>0 Have not had in the past 30 days<br>1 Once a month

			2 A couple of days a month<br>3 Once a week<br>4 A couple of days a week<br>5 Everyday<br>99 Don't know or prefer not to answer
234	gen_last4wk_b	Bloating	radio (Matrix)<br>0 Have not had in the past 30 days<br>1 Once a month<br>2 A couple of days a month<br>3 Once a week<br>4 A couple of days a week<br>5 Everyday<br>99 Don't know or prefer not to answer
235	gen_last4wk_c	Breast Tenderness	radio (Matrix)<br>0 Have not had in the past 30 days<br>1 Once a month<br>2 A couple of days a month<br>3 Once a week<br>4 A couple of days a week<br>5 Everyday<br>99 Don't know or prefer not to answer
236	gen_last4wk_d	Moodiness or irritability	radio (Matrix)<br>0 Have not had in the past 30 days<br>1 Once a month<br>2 A couple of days a month<br>3 Once a week<br>4 A couple of days a week<br>5 Everyday<br>99 Don't know or prefer not to answer
237	gen_last4wk_e	Acne flare-up	radio (Matrix)<br>0 Have not had in the past 30 days<br>1 Once a month<br>2 A couple of days a month<br>3 Once a week<br>4 A couple of days a week<br>5 Everyday<br>99 Don't know or prefer not to answer
238	gen_last4wk_f	Cramping	radio (Matrix)<br>0 Have not had in the past 30 days<br>1 Once a month<br>2 A couple of days a month<br>3 Once a week<br>4 A couple of days a week

			5 Everyday<br>99 Don't know or prefer not to answer
239	gen_last4wk_g	Weight Gain	radio (Matrix)<br>0 Have not had in the past 30 days<br>1 Once a month<br>2 A couple of days a month<br>3 Once a week<br>4 A couple of days a week<br>5 Everyday<br>99 Don't know or prefer not to answer
240	gen_last4wk_h	Weight Loss	radio (Matrix)<br>0 Have not had in the past 30 days<br>1 Once a month<br>2 A couple of days a month<br>3 Once a week<br>4 A couple of days a week<br>5 Everyday<br>99 Don't know or prefer not to answer
241	gen_last4wk_i	Depressed mood	radio (Matrix)<br>0 Have not had in the past 30 days<br>1 Once a month<br>2 A couple of days a month<br>3 Once a week<br>4 A couple of days a week<br>5 Everyday<br>99 Don't know or prefer not to answer
242	gen_last4wk_j	Constipation or diarrhea	radio (Matrix)<br>0 Have not had in the past 30 days<br>1 Once a month<br>2 A couple of days a month<br>3 Once a week<br>4 A couple of days a week<br>5 Everyday<br>99 Don't know or prefer not to answer
243	pms_care<br>Show the field ONLY if:<br>[gen_last4wk_a] = '1' or [gen_last4wk_a] = '2' or [gen_last4wk_a] = '3' or [gen_last4wk_a] = '4' or [gen_last4wk_a] = '5' or [gen_last4wk_b] = '1' or [gen_last4wk_b] = '2' or [gen_last4wk_b] = '3' or [gen_last4wk_b] = '4' or [gen_last4wk_b] = '5' or [gen_last4wk_c] = '1' or [gen_last4wk_c] = '2' or [gen_last4wk_c] = '3' or [gen_last4wk_c] = '	Have you sought medical care for any of these conditions?	radio<br>0 No<br>1 Yes<br>88 Don't know

	4' or [gen_last4wk_c] = '5' or [gen_last4wk_d] = '1' or [gen_last4wk_d] = '2' or [gen_last4wk_d] = '3' or [gen_last4wk_d] = '4' or [gen_last4wk_d] = '5' or [gen_last4wk_e] = '1' or [gen_last4wk_e] = '2' or [gen_last4wk_e] = '3' or [gen_last4wk_e] = '4' or [gen_last4wk_e] = '5' or [gen_last4wk_f] = '1' or [gen_last4wk_f] = '2' or [gen_last4wk_f] = '3' or [gen_last4wk_f] = '4' or [gen_last4wk_f] = '5' or [gen_last4wk_g] = '1' or [gen_last4wk_g] = '2' or [gen_last4wk_g] = '3' or [gen_last4wk_g] = '4' or [gen_last4wk_g] = '5' or [gen_last4wk_h] = '1' or [gen_last4wk_h] = '2' or [gen_last4wk_h] = '3' or [gen_last4wk_h] = '4' or [gen_last4wk_h] = '5' or [gen_last4wk_i] = '1' or [gen_last4wk_i] = '2' or [gen_last4wk_i] = '3' or [gen_last4wk_i] = '4' or [gen_last4wk_i] = '5' or [gen_last4wk_j] = '1' or [gen_last4wk_j] = '2' or [gen_last4wk_j] = '3' or [gen_last4wk_j] = '4' or [gen_last4wk_j] = '5'		
244	pms_care_what<br>Show the field ONLY if:<br>[pms_care] = '1'	If so, please specify which one(s):	text
245	pms_rx<br>Show the field ONLY if:<br>[pms_care] = '1'	Were you prescribed medication to treat these conditions?	radio<br>0 No<br>1 Yes
246	sexsat_scale	Section Header: *Next, we want to gather information about your sexual relationships. Please remember that everything you say is confidential.*<br>On a scale of 1 to 100, how might you rank your sex life right now?	slider (number)<br>Slider labels: worst possible, , best possible<br>Custom alignment: RH
247	impact_sexlife	In the last 4 weeks, would you say that your birth control or method to avoid pregnancy has:	radio<br>2 Improved my sex life a lot<br>1 Improved my sex life a little<br>0 Has had no effect on my sex life<br>-1 Has made my sex life a little worse<br>-2 Has made my sex life a lot worse
248	impact_sexlife_txt	Briefly explain the impact your method used in the last 4 weeks to avoid pregnancy has on your sex life.	notes
249	currently_sexual	Have you been sexually active with a male partner in the past four weeks? This may include a variety of activities, not just vaginal intercourse.	radio, Required<br>0 No<br>1 Yes<br>99 I prefer not to answer
250	relationship_length<br>Show the field ONLY if:<br>[currently_sexual] = '1'	How long have you been in your sexual relationship?<br><br>Note: If you have more than one partner, think about your primary/main partner when answering.	radio<br>1 Less than 3 months<br>2 3 to 6 months<br>3 6 months to 1 year

			4 1 to 2 years<br>5 2 to 3 years<br>6 More than 3 years
251	relationship_yrs<br>Show the field ONLY if:<br>[relationship_length] = '6'	If you have been in a relationship with your primary sexual partner for more than 3 years, please tell us how many years it has been.<br>*years*	text
252	fsfi_desire_level	Section Header: *We will now ask you a few questions about your sexual feelings and responses during the past four weeks.*<br>Over the past 4 weeks, how would you rate your level (degree) of sexual desire or interest?	radio, Required<br>5 Very high<br>4 High<br>3 Moderate<br>2 Low<br>1 Very low or none at all<br>99 Prefer not to answer
253	fsfi_arousal_level	Over the past 4 weeks, how would you rate your level of sexual arousal ("turn on") during sexual activity or intercourse?	radio, Required<br>0 No sexual activity<br>5 Very high<br>4 High<br>3 Moderate<br>2 Low<br>1 Very low or none at all<br>99 Prefer not to answer
254	fsfi_lube_freq	Over the past 4 weeks, how often did you become lubricated ("wet") during sexual activity or intercourse?	radio, Required<br>0 No sexual activity<br>5 Almost always or always<br>4 Most times (more than half the time)<br>3 Sometimes (about half the time)<br>2 A few times (less than half the time)<br>1 Almost never or never<br>99 Prefer not to answer
255	fsfi_orgasm_freq	Over the past 4 weeks, when you had sexual stimulation or intercourse, how often did you reach orgasm (climax)?	radio, Required<br>0 No sexual activity<br>5 Almost always or always<br>4 Most times (more than half the time)<br>3 Sometimes (about half the time)<br>2 A few times (less than half the time)<br>1 Almost never or never<br>99 Prefer not to answer
256	fsfi_overall_sat	Over the past 4 weeks, how satisfied have you been with your overall sexual life?	radio, Required<br>5 Very satisfied<br>4 Moderately satisfied<br>3 About equally satisfied and dissatisfied<br>2 Moderately dissatisfied<br>1 Very dissatisfied

			99 Prefer not to answer
257	fsfi_pain_freq	Over the past 4 weeks, how often did you experience discomfort or pain during vaginal penetration?	radio, Required<br>0 Did not attempt intercourse<br>1 Almost always or always<br>2 Most times (more than half the time)<br>3 Sometimes (about half the time)<br>4 A few times (less than half the time)<br>5 Almost never or never<br>99 Prefer not to answer
258	nsss_intensity	Section Header: *The following items relate to your sexual experiences. You might find a few of the items similar to items you just filled out. That's okay; just do your best to answer all the questions. When responding to these items, please think about the last 4 weeks. Thinking about the last month, how satisfied or dissatisfied are you with each of the following issues?*<br>The intensity of my sexual arousal	radio (Matrix), Required<br>0 not at all satisfied<br>1 a little satisfied<br>2 moderately satisfied<br>3 very satisfied<br>4 extremely satisfied<br>99 I prefer not to answer
259	nsss_orgasm	The quality of my orgasms	radio (Matrix), Required<br>0 not at all satisfied<br>1 a little satisfied<br>2 moderately satisfied<br>3 very satisfied<br>4 extremely satisfied<br>99 I prefer not to answer
260	nsss_selfsurreder	My letting go and surrender to sexual pleasure during sex	radio (Matrix), Required<br>0 not at all satisfied<br>1 a little satisfied<br>2 moderately satisfied<br>3 very satisfied<br>4 extremely satisfied<br>99 I prefer not to answer
261	nsss_concentrate	My focus and concentration during sexual activity	radio (Matrix), Required<br>0 not at all satisfied<br>1 a little satisfied<br>2 moderately satisfied<br>3 very satisfied<br>4 extremely satisfied<br>99 I prefer not to answer
262	nsss_react	The way I sexually react to my partner	radio (Matrix), Required<br>0 not at all satisfied<br>1 a little satisfied<br>2 moderately satisfied<br>3 very satisfied<br>4 extremely satisfied

			99 I prefer not to answer
263	nsss_function	My body's sexual functioning	radio (Matrix), Required<br>0 not at all satisfied<br>1 a little satisfied<br>2 moderately satisfied<br>3 very satisfied<br>4 extremely satisfied<br>99 I prefer not to answer
264	nsss_emotion	My emotional opening up in sex	radio (Matrix), Required<br>0 not at all satisfied<br>1 a little satisfied<br>2 moderately satisfied<br>3 very satisfied<br>4 extremely satisfied<br>99 I prefer not to answer
265	nsss_moodafter	My mood after sexual activity	radio (Matrix), Required<br>0 not at all satisfied<br>1 a little satisfied<br>2 moderately satisfied<br>3 very satisfied<br>4 extremely satisfied<br>99 I prefer not to answer
266	nsss_orgasmfreq	The frequency of my orgasms	radio (Matrix), Required<br>0 not at all satisfied<br>1 a little satisfied<br>2 moderately satisfied<br>3 very satisfied<br>4 extremely satisfied<br>99 I prefer not to answer
267	nsss_givepleasure	The pleasure I provide to my partner	radio (Matrix), Required<br>0 not at all satisfied<br>1 a little satisfied<br>2 moderately satisfied<br>3 very satisfied<br>4 extremely satisfied<br>99 I prefer not to answer
268	nsss_balance	The balance between what I give and receive in sex	radio (Matrix), Required<br>0 not at all satisfied<br>1 a little satisfied<br>2 moderately satisfied<br>3 very satisfied<br>4 extremely satisfied<br>99 I prefer not to answer

269	nsss_pt_emotion	My partner's emotional opening up during sex	radio (Matrix), Required<br>0 not at all satisfied<br>1 a little satisfied<br>2 moderately satisfied<br>3 very satisfied<br>4 extremely satisfied<br>99 I prefer not to answer
270	nsss_pt_initiation	My partner's initiation of sexual activity	radio (Matrix), Required<br>0 not at all satisfied<br>1 a little satisfied<br>2 moderately satisfied<br>3 very satisfied<br>4 extremely satisfied<br>99 I prefer not to answer
271	nsss_pt_orgasm	My partner's ability to orgasm	radio (Matrix), Required<br>0 not at all satisfied<br>1 a little satisfied<br>2 moderately satisfied<br>3 very satisfied<br>4 extremely satisfied<br>99 I prefer not to answer
272	nsss_pt_surrender	My partner's surrender to sexual pleasure (letting go)	radio (Matrix), Required<br>0 not at all satisfied<br>1 a little satisfied<br>2 moderately satisfied<br>3 very satisfied<br>4 extremely satisfied<br>99 I prefer not to answer
273	nsss_pt_care	The way my partner takes care of my sexual needs	radio (Matrix), Required<br>0 not at all satisfied<br>1 a little satisfied<br>2 moderately satisfied<br>3 very satisfied<br>4 extremely satisfied<br>99 I prefer not to answer
274	nsss_pt_creativity	My partner's sexual creativity	radio (Matrix), Required<br>0 not at all satisfied<br>1 a little satisfied<br>2 moderately satisfied<br>3 very satisfied<br>4 extremely satisfied<br>99 I prefer not to answer
275	nsss_pt_availability	My partner's sexual availability	radio (Matrix), Required

			0 not at all satisfied<br>1 a little satisfied<br>2 moderately satisfied<br>3 very satisfied<br>4 extremely satisfied<br>99 I prefer not to answer
276	nsss_variety	The variety of my sexual activities	radio (Matrix), Required<br>0 not at all satisfied<br>1 a little satisfied<br>2 moderately satisfied<br>3 very satisfied<br>4 extremely satisfied<br>99 I prefer not to answer
277	nsss_frequency	The frequency of my sexual activity	radio (Matrix), Required<br>0 not at all satisfied<br>1 a little satisfied<br>2 moderately satisfied<br>3 very satisfied<br>4 extremely satisfied<br>99 I prefer not to answer
278	sf_concern	Do you have any concern about your sexual functioning?	radio, Required<br>0 No<br>1 Yes<br>99 I don't know
279	sf_consern_txt<br>Show the field ONLY if:<br>[sf_concern] = '1' or [sf_concern] = '99'	Please briefly describe.	notes
280	who_1	Section Header: *The last few items have to do with more general health and well-being. Please think about your experiences in the last four weeks when answering.*<br>I have felt cheerful and in good spirits	radio (Matrix), Required<br>5 All of the time<br>4 Most of the time<br>3 More than half of the time<br>2 Less than half of the time<br>1 Some of the time<br>0 At no time
281	who_2	I have felt calm and relaxed	radio (Matrix), Required<br>5 All of the time<br>4 Most of the time<br>3 More than half of the time<br>2 Less than half of the time<br>1 Some of the time<br>0 At no time
282	who_3	I have felt active and vigorous	radio (Matrix), Required

			5 All of the time<br>4 Most of the time<br>3 More than half of the time<br>2 Less than half of the time<br>1 Some of the time<br>0 At no time
283	who_4	I woke up feeling fresh and rested	radio (Matrix), Required<br>5 All of the time<br>4 Most of the time<br>3 More than half of the time<br>2 Less than half of the time<br>1 Some of the time<br>0 At no time
284	who_5	My daily life has been filled with things that interest me	radio (Matrix), Required<br>5 All of the time<br>4 Most of the time<br>3 More than half of the time<br>2 Less than half of the time<br>1 Some of the time<br>0 At no time
285	future_studies	Would you be willing to have study staff contact you for future studies?	yesno<br>1 Yes<br>0 No
286	comments	Is there anything else you would like us to know?	notes
287	enrollment_survey_complete	Section Header: *Form Status*<br>Complete?	dropdown<br>0 Incomplete<br>1 Unverified<br>2 Complete

Instrument: **Followup Survey (1, 3, & 6 months)** (followup_survey_1_3_6_months) Enabled as survey Expand

Instrument: **Brief Followup Survey (1, 2, & 3 years)** (brief_followup_survey_1_2_3_years) Enabled as survey Collapse

375	contact_chng_yr	Section Header: *Thank you very much for participating in this study. First we want to make sure you haven't changed any of your contact information.*<br>In the past six months, has any of your contact information changed (that is, your phone number, email, or mailing address)?	radio<br>0 No<br>1 Yes
376	which_contact_chng_yr<br>Show the field ONLY if:<br>[contact_chng_yr] = '1'	If so, which contact info has changed?<br>*check all that apply*	checkbox<br>1 which_contact_chng_yr___1 New phone number<br>2 which_contact_chng_yr___2 New email address<br>3 which_contact_chng_yr___3 New home address
377	new_phone_yr<br>Show the field ONLY if:<br>[which_contact_chng_yr(1)] = '1'	Please enter in your new phone number.<br>*xxx-xxx-xxxx*	text (phone), Identifier
378	new_email_yr	Please enter in your new email address.	text (email), Identifier

	Show the field ONLY if: [which_contact_chng_yr(2)] = '1'		
379	new_addy_yr<br>Show the field ONLY if: [which_contact_chng_yr(3)] = '1'	Please enter in your new mailing address.	notes, Identifier
380	bc_recent_yr	Section Header: *The following questions are about your pregnancy plans and recent changes in contraception.*<br>What method(s) to prevent pregnancy have you used in the last 4 weeks?<br>*select all that apply*	checkbox<br>1 bc_recent_yr___1 Contraceptive Implant (Nexplanon)<br>2 bc_recent_yr___2 Copper IUD (Paragard)<br>3 bc_recent_yr___3 Hormonal IUD (Mirena)<br>4 bc_recent_yr___4 Hormonal IUD (Liletta)<br>5 bc_recent_yr___5 Other hormonal IUD (Skyla)<br>6 bc_recent_yr___6 Injection (Depo-Provera)<br>7 bc_recent_yr___7 Combined oral contraceptive pill (The Pill)<br>8 bc_recent_yr___8 Progestin Only Pill (Minipill)<br>9 bc_recent_yr___9 Contraceptive patch (Xulane or OrthoEvra)<br>10 bc_recent_yr___10 Vaginal ring (NuvaRing)<br>11 bc_recent_yr___11 Male condom<br>12 bc_recent_yr___12 Female condom<br>13 bc_recent_yr___13 Cervical cap or sponge<br>14 bc_recent_yr___14 Spermicide<br>15 bc_recent_yr___15 Diaphragm<br>16 bc_recent_yr___16 Fertility Awareness Method / Natural Family Planning / Rhythm Method<br>17 bc_recent_yr___17 Withdrawal<br>18 bc_recent_yr___18 Levonorgestrel Emergency Contraception (Plan B/Next Choice)<br>19 bc_recent_yr___19 Ulipristal EC Pill (Ella)<br>99 bc_recent_yr___99 Other<br>0 bc_recent_yr___0 None; I am not using any contraceptive method
381	other_bc_last_sex_yr<br>Show the field ONLY if: [bc_recent_yr(99)] = '1'	If other, please specify:	text
382	herc_still_yr<br>Show the field ONLY if: [contraception_still_mo] = '0' or [contraception_still_midyr] = '0'	Are you still using the birth control method that you received at the beginning of this study?	radio<br>0 No<br>1 Yes
383	herc_selfcheck_yr<br>Show the field ONLY if: [bc_recent_yr(1)] = '1' or [bc_r	In the last 4 weeks, have you checked to make sure your IUD or implant is still in place?	radio<br>0 No, I have not<br>1 Yes, I felt my strings (or rod in my arm) myself

	ecent_yr(2)] = '1' or [bc_recent_yr(3)] = '1' or [bc_recent_yr(4)] = '1' or [bc_recent_yr(5)] = '1'		and confirmed it's still there<br>2 Yes, I had a provider check<br>3 I tried but was not able to feel anything
384	iud_length_yr<br>Show the field ONLY if:<br>[bc_recent_yr(1)] = '1' or [bc_recent_yr(2)] = '1' or [bc_recent_yr(3)] = '1' or [bc_recent_yr(4)] = '1' or [bc_recent_yr(5)] = '1' or [bc_recent_yr(6)] = '1' or [bc_recent_yr(7)] = '1' or [bc_recent_yr(8)] = '1' or [bc_recent_yr(9)] = '1' or [bc_recent_yr(10)] = '1' or [bc_recent_yr(11)] = '1' or [bc_recent_yr(12)] = '1' or [bc_recent_yr(13)] = '1' or [bc_recent_yr(14)] = '1' or [bc_recent_yr(15)] = '1' or [bc_recent_yr(16)] = '1' or [bc_recent_yr(17)] = '1' or [bc_recent_yr(18)] = '1' or [bc_recent_yr(19)] = '1' or [bc_recent_yr(99)] = '1'	How long do you plan on using the birth control method you are using?	radio<br>1 Less than 1 year<br>2 More than 1 year but less than 2 years<br>3 More than 2 years but less than 3 years<br>4 More than 3 years but less than 5 years<br>5 More than 5 years but less than 10 years<br>6 More than 10 years<br>7 Unsure<br>99 I prefer not to answer this question
385	removal_reason_yr<br>Show the field ONLY if:<br>[herc_still_yr] = '0'	What are the reasons you are no longer using the method you started at the beginning of the study?	checkbox<br>1 removal_reason_yr___1 Bleeding issues (spotting, irregular, ect)<br>2 removal_reason_yr___2 Excessive bleeding<br>3 removal_reason_yr___3 Cramping<br>4 removal_reason_yr___4 Pain<br>5 removal_reason_yr___5 Breast symptoms<br>6 removal_reason_yr___6 Weight gain<br>7 removal_reason_yr___7 Weight loss<br>8 removal_reason_yr___8 Moodiness or depression<br>9 removal_reason_yr___9 Bloating<br>10 removal_reason_yr___10 Skin problems<br>16 removal_reason_yr___16 Loss of libido/sexual interest<br>11 removal_reason_yr___11 Pain during intercourse<br>12 removal_reason_yr___12 Partner complaint<br>13 removal_reason_yr___13 IUD fell out/expelled<br>14 removal_reason_yr___14 I wanted to get pregnant<br>15 removal_reason_yr___15 I had a positive pregnancy test<br>99 removal_reason_yr___99 Other
386	removal_reason_oth_yr<br>Show the field ONLY if:<br>[removal_reason_yr(99)] = '1'	If other, please specify:	text
387	herc_removal_date_yr	What was the date the device fell out or was removed?	text (date_mdy)

	Show the field ONLY if:<br>[herc_still_yr] = '0'	If you can't remember the exact date, just make your best guess.	
388	switch_date_yr<br>Show the field ONLY if:<br>[herc_still_yr] = '0' and [bc_recent_mo(0)] = '0'	When did you start using your new method?<br>*please make you best guess*	text
389	sat_main_bc_yr	Overall, how satisfied are you with the method(s) you were using during the last 4 weeks?	radio, Required<br>1 Completely satisfied<br>2 Somewhat satisfied<br>3 Neither satisfied or dissatisfied<br>4 Somewhat dissatisfied<br>5 Completely dissatisfied<br>99 I prefer not to answer
390	confidence_bc_yr	Overall, how confident were you that the method(s) you have been using the last 4 weeks will prevent pregnancy?	radio, Required<br>5 Very high confidence<br>4 High confidence<br>3 Moderate confidence<br>2 Low confidence<br>1 Very low or no confidence<br>99 I prefer not to answer
391	control_preg_yr	Please rate your agreement or disagreement with the following statement: "I feel that I have control over whether or not I get pregnant."	radio, Required<br>1 I strongly agree<br>2 I somewhat agree<br>3 I neither agree nor disagree<br>4 I somewhat disagree<br>5 I strongly disagree<br>99 I prefer not to answer
392	impact_sexlife_yr	In the last 4 weeks, would you say that your birth control or method to avoid pregnancy has:	radio<br>2 Improved my sex life a lot<br>1 Improved my sex life a little<br>0 Has had no effect on my sex life<br>-1 Has made my sex life a little worse<br>-2 Has made my sex life a lot worse
393	impact_sexlife_txt_yr	Briefly explain the impact your method used in the last 4 weeks to avoid pregnancy has on your sex life.	notes
394	future_children_yr	Section Header: *Now we would like to ask you about your plans for future children, ideal timing, and feelings surrounding pregnancy.*<br>Do you think you would like to have children (or more children) in the future?	radio<br>0 No<br>1 Yes<br>99 I don't know
395	preg_plans_yr	What are your future pregnancy plans?	radio<br>0 I do not plan on getting pregnant at any time in the future.<br>1 I am currently trying to get pregnant.<br>2 I would like to get pregnant in the next year.<br>3 I would like to get pregnant in the next 2-5

			years (but not this year).<br>4 I would like to get pregnant in the next 5-10 years but not before then.<br>88 Unsure<br>99 Other
396	preg_plans_oth_yr<br>Show the field ONLY if:<br>[preg_plans_yr] = '99'	What are your pregnancy plans?	text
397	preg_intention_import_yr<br>Show the field ONLY if:<br>[future_children_yr] >= '1'	How important is it to you to not get pregnant until you are ready?	slider<br>Slider labels: not at all important, , the most important<br>Custom alignment: RH
398	preg_avoid_impor_yr<br>Show the field ONLY if:<br>[future_children_yr] = '0'	How important is it to you to not get pregnant now or in the future?	slider<br>Slider labels: not at all important, , the most important<br>Custom alignment: RH
399	preg_happy_yr	How would you feel if you got pregnant in the next month?	slider<br>Slider labels: worst feeling you can imagine, , happiest you could possibly feel<br>Custom alignment: RH
400	preg_impact_yr	Please tell us a bit more about how a pregnancy now or in the near future would affect your life.	notes
401	religion_2	Which of the following best describes your religious identity?	radio<br>0 Not Religious<br>1 Christian (Protestant, Evangelical, Mainline, etc)<br>2 Catholic<br>3 Mormon<br>4 Jewish<br>5 Muslim<br>6 Other<br>88 Don't know or prefer not to answer
402	religion_txt_2<br>Show the field ONLY if:<br>[religion] = '6'	Please describe your religious identity.	text
403	gender_id_yr	Section Header: *Please tell us a bit more about yourself. None of your answers will impact any services you may receive.*<br>We understand that not everyone who needs or wants birth control identifies as a woman.<br><br>Please select the word(s) you use to describe yourself, or select self-describe and you can write in the word(s) you use.<br>*check all that apply*	checkbox<br>1 gender_id_yr___1 Woman<br>2 gender_id_yr___2 Man<br>3 gender_id_yr___3 Non-binary<br>4 gender_id_yr___4 Transgender<br>5 gender_id_yr___5 Prefer to self-describe
404	gender_txt_yr<br>Show the field ONLY if:<br>[gender_id_yr(5)] = '1'	Please describe yourself.	text
405	sexual_identitiy2_yr	Please choose the description that best fits how you think of yourself.	radio, Required<br>1 Exclusively Heterosexual (Straight)<br>5 Mostly Heterosexual<br>3 Bisexual (Attracted to both Men and Women)<br>6 Mostly Gay/Lesbian

			2 Exclusively Homosexual (Gay/Lesbian)<br>7 Not sexually attracted to either males or females<br>4 Other<br>88 I don't know<br>99 I prefer not to answer<br><br>Field Annotation: The categories of mostly heterosexual and mostly gay as well as not sexually attracted to either were added 2/25/16 per Bethany Everett recomendations
406	sexwith2_yr	In the last 12 months have you had sex with...	radio<br>0 Not currently sexually active<br>1 Males only<br>2 Both males and females<br>3 Females only<br>88 I prefer not to answer
407	relationship_yr	Which of the following best describes your current relationship situation?	radio<br>1 Married<br>2 Not married, but living together or in a committed relationship<br>3 Actively dating, but NOT in a committed relationship<br>4 Divorced/Separated<br>5 Single, not in a relationship<br>6 Widowed<br>7 Other<br>99 I prefer not to answer
408	insurance_yr	What type of medical insurance do you currently have?	radio, Required<br>1 None<br>2 Medicaid<br>4 Insurance through your job or that you buy on your own<br>6 Student health insurance<br>7 Parent's insurance<br>5 Military or VA (Champus, ChampVA, Tricare)<br>3 Disability or Medicare<br>88 I don't know<br>99 I prefer not to answer
409	employment_yr	What best describes your current employment status?	checkbox, Required<br>0 employment_yr___0 Unemployed<br>1 employment_yr___1 Working full-time (at least 30 hours/week)<br>2 employment_yr___2 Working part-time (less than 30 hours/week)<br>3 employment_yr___3 Disabled, sick leave, family leave

			4 employment_yr___4 Retired<br>5 employment_yr___5 Homemaker<br>6 employment_yr___6 Student<br>8 employment_yr___8 Other<br>99 employment_yr___99 I prefer not to answer
410	employment_oth_yr<br>Show the field ONLY if:<br>[employment_yr(8)] = '1'	If other employment, please describe.	text
411	employ_hours_yr<br>Show the field ONLY if:<br>[employment_yr(1)] = '1' or [employment_yr(2)] = '1'	How many hours do you work each week?<br>*Please guess if you are not sure*	text (number)
412	hourly_wage_yr<br>Show the field ONLY if:<br>[employment_yr(1)] = '1' or [employment_yr(2)] = '1'	What is your hourly wage?	text
413	employ_type_yr<br>Show the field ONLY if:<br>[employment_yr(1)] = '1' and [employment_yr(2)] = '1'	What kind of work do you do?	notes
414	lookingforwork_yr	Are you currently looking for work, additional work, or different work?	radio<br>0 No<br>1 Yes<br>3 Don't Know<br>4 I prefer not to answer this question
415	jail_yn	Were you incarcerated in jail or prison in the last 12 months? (Even if only for a few hours or days)	radio<br>0 No<br>1 Yes<br>99 I prefer not to answer
416	jail_number<br>Show the field ONLY if:<br>[jail_yn] = '1'	How many times were you incarcerated in the last 12 months? (Even if only for a few hours or days)	dropdown (autocomplete)<br>1 1<br>2 2<br>3 3<br>4 4<br>5 5<br>6 6<br>7 7<br>8 8<br>9 9<br>10 10<br>11 11<br>12 12 or more
417	jail_dates<br>Show the field ONLY if:<br>[jail_yn] = '1'	Please list the dates you were incarcerated (your best guess is fine) and where.<br><br>example<br>1) 1/15/16-1/20/16, Salt Lake County Jail	notes

		2) 2/5/16-4/5/16, Utah State Prision	
418	jail_nobc<br>Show the field ONLY if:<br>[jail_yn] = '1'	Were you able to continue using your birth control while you were incarcerated?	yesno<br>1 Yes<br>0 No
419	jail_txt<br>Show the field ONLY if:<br>[jail_yn] = '1'	Please describe the ways your incarceration impacted your use of birth control or pregnancy plans.	notes
420	education_yr	Section Header: *The next few questions are about your education.*<br>What best describes the highest level of education you have COMPLETED SO FAR?	radio, Required<br>1 11th grade or less<br>2 12th grade (completed high school or GED)<br>3 Vocational/technical training<br>4 Associate degree or some college<br>5 4-year college degree (BA/BS)<br>6 Any graduate or professional education (any time in a Masters, JD, PhD, MD, etc. program)<br>88 I don't know<br>99 I prefer not to answer
421	inschool_yr	Are you currently in school, either full-time or part-time?	radio<br>0 Not at all<br>1 Part-time<br>2 Full-time<br>3 I prefer not to answer this question
422	student_degree_yr<br>Show the field ONLY if:<br>[inschool_yr] = '1' or [inschool_yr] = '2'	What type of degree are you seeking?	radio<br>1 I plan to finish high school or GED<br>2 Get vocational/technical training<br>3 Get an associate degree<br>4 Get a 4-year college degree (BA/BS)<br>5 Get graduate or professional education (Masters, JD PhD, MD)<br>88 I don't know<br>99 I prefer not to answer
423	degree_type_yr<br>Show the field ONLY if:<br>[student_degree_yr] = '2' or [student_degree_yr] = '3' or [student_degree_yr] = '4' or [student_degree_yr] = '5'	Please specify type	text
424	student_grddate_yr<br>Show the field ONLY if:<br>[student_degree_yr] = '1' or [student_degree_yr] = '2' or [student_degree_yr] = '3' or [student_degree_yr] = '4' or [student_degree_yr] = '5'	What date do you expect to graduate?	text (date_mdy)
425	education_plans_yr	What best describes your PLANS for the highest level of education you hope to achieve in the future?	radio, Required<br>0 None, I am done with school and do not have plans to go back

			1 I plan to finish high school or GED<br>2 Get vocational/technical training<br>3 Get an associate degree<br>4 Get a 4-year college degree (BA/BS)<br>5 Get graduate or professional education (Masters, JD PhD, MD)<br>88 I don't know<br>99 I prefer not to answer
426	income_annual_yr	Section Header: *Just a few more questions about your financial situation.*<br>What is your annual household income?	radio, Required<br>1 Less than $10,000<br>2 $10,000-$19,999<br>3 $20,000-$29,999<br>4 $30,000-$39,999<br>5 $40,000-$49,999<br>6 $50,000-$59,999<br>7 $60,000-$69,999<br>8 $70,000-$79,999<br>9 $80,000 or more<br>10 I don't know<br>11 I prefer not to answer this question
427	income_annual_exact_yr	What is your best estimate of your annual household income?	text
428	dependents_yr	How many people are in your household?	dropdown<br>1 1<br>2 2<br>3 3<br>4 4<br>5 5<br>6 6<br>7 7<br>8 8<br>9 9<br>10 10<br>11 11<br>12 12<br>13 13<br>14 14<br>15 15+
429	children_yr	How many children under the age of 18 do you have living with you?	text
430	incomesource_yr	Please check all of the following that have been a source of income in the last month:<br>*Check all that apply*	checkbox<br>1 incomesource_yr___1 Self<br>2 incomesource_yr___2 Spouse or Partner<br>3 incomesource_yr___3 Other family member<br>4 incomesource_yr___4 Government assistance

			5 incomesource_yr___5 Other<br>6 incomesource_yr___6 I prefer not to answer
431	oth_income_txt_yr<br>Show the field ONLY if:<br>[incomesource_yr(5)] = '1'	What other sources of income did you receive in the last month?	text
432	income_mo_yr<br>Show the field ONLY if:<br>[incomesource_yr(1)] = '1'	How much money did you make last month?	radio, Required<br>1 None<br>2 $1-$400<br>3 $401-$800<br>4 $801-$1,200<br>5 $1,201-$1,600<br>6 $1,601-$2,000<br>7 $2,001-$2,400<br>8 $2,401-$2,800<br>9 More than $2,800<br>88 Don't know<br>99 I prefer not to answer
433	pt_income_mo_yr<br>Show the field ONLY if:<br>[incomesource_yr(2)] = '1'	How much money did your partner or spouse make last month?	radio<br>1 None<br>2 $1-$400<br>3 $401-$800<br>4 $801-$1,200<br>5 $1,201-$1,600<br>6 $1,601-$2,000<br>7 $2,001-$2,400<br>8 $2,401-$2,800<br>9 More than $2,800<br>88 Don't know<br>99 I prefer not to answer
434	fam_income_mo_yr<br>Show the field ONLY if:<br>[incomesource_yr(3)] = '1'	How much money did your other family members contribute to your household income last month?	radio<br>1 None<br>2 $1-$400<br>3 $401-$800<br>4 $801-$1,200<br>5 $1,201-$1,600<br>6 $1,601-$2,000<br>7 $2,001-$2,400<br>8 $2,401-$2,800<br>9 More than $2,800<br>88 Don't know<br>99 I prefer not to answer
435	gov_income_mo_yr<br>Show the field ONLY if:	How much money did government assistance contribute to your household income last month?	radio<br>1 None

	[incomesource_yr(4)] = '1'		2 | $1-$400<br>3 | $401-$800<br>4 | $801-$1,200<br>5 | $1,201-$1,600<br>6 | $1,601-$2,000<br>7 | $2,001-$2,400<br>8 | $2,401-$2,800<br>9 | More than $2,800<br>88 | Don't know<br>99 | I prefer not to answer
436	oth_income_mo_yr<br>Show the field ONLY if:<br>[incomesource_yr(5)] = '1'	How much money did you receive from other sources last month?	radio<br>1 | None<br>2 | $1-$400<br>3 | $401-$800<br>4 | $801-$1,200<br>5 | $1,201-$1,600<br>6 | $1,601-$2,000<br>7 | $2,001-$2,400<br>8 | $2,401-$2,800<br>9 | More than $2,800<br>88 | Don't know<br>99 | I prefer not to answer
437	childsupport_planned_yr<br>Show the field ONLY if:<br>[dependents_yr] >= 1	Were you supposed to receive any child support in the last 4 weeks?	radio<br>0 | No<br>1 | Yes<br>2 | I prefer not to answer
438	childsupport_amt_yr<br>Show the field ONLY if:<br>[childsupport_actual_yr] = '1'	How much child support were you supposed to receive?	text
439	childsupport_actual_yr<br>Show the field ONLY if:<br>[dependents_yr] >= 1 or [child support_planned_yr] ='1'	Did you receive any child support last 4 weeks?	radio<br>0 | No<br>1 | Yes<br>3 | I prefer not to answer this question
440	childsupport_amtactual_yr<br>Show the field ONLY if:<br>[childsupport_actual_yr] = '1'	How much child support did you receive in the last 4 weeks?	text
441	typehome_yr	Which of the following best describes your current housing situation?	radio<br>0 | Homeless<br>1 | Shelter<br>2 | Mobile home<br>3 | Apartment<br>4 | Single-family house<br>6 | Other<br>9 | I prefer not to answer this question

442	otherhousing_yr<br>Show the field ONLY if:<br>[typehome_yr] = '6'	Describe type of housing:	text
443	asst_food_yr	Section Header: *We will now ask a few questions about public assistance you may receive: Do you currently receive:*<br>Food stamps	radio (Matrix), Required<br>0 No<br>1 Yes<br>99 Prefer not to answer
444	asst_wic_yr	WIC (Women, Infants and Children)	radio (Matrix), Required<br>0 No<br>1 Yes<br>99 Prefer not to answer
445	asst_welfare_yr	Welfare	radio (Matrix), Required<br>0 No<br>1 Yes<br>99 Prefer not to answer
446	asst_unemployment_yr	Unemployment benefits	radio (Matrix), Required<br>0 No<br>1 Yes<br>99 Prefer not to answer
447	pay_trans_yr	Section Header: *Just a few more questions about your economic situation. During the past 12 months, have you had trouble paying for the following:*<br>Transportation	radio (Matrix), Required<br>0 No<br>1 Yes<br>99 Prefer not to answer
448	pay_housing_yr	Housing	radio (Matrix), Required<br>0 No<br>1 Yes<br>99 Prefer not to answer
449	pay_health_yr	Medical care or medications	radio (Matrix), Required<br>0 No<br>1 Yes<br>99 Prefer not to answer
450	pay_food_yr	Food	radio (Matrix), Required<br>0 No<br>1 Yes<br>99 Prefer not to answer
451	enoughmoney_yr	During the past month, how often would you say you had enough money to meet your basic living needs such as food, housing and transportation?	radio<br>1 All the time<br>2 Most of the time<br>3 Some of the time<br>4 Rarely<br>5 Never<br>6 Don't Know<br>7 Refused

452	who_1_yr	Section Header: *The last few items have to do with your general health and well-being. Please think about the last 4 weeks when responding to these items.*<br>I have felt cheerful and in good spirits	radio (Matrix), Required<br>5 All of the time<br>4 Most of the time<br>3 More than half of the time<br>2 Less than half of the time<br>1 Some of the time<br>0 At no time
453	who_2_yr	I have felt calm and relaxed	radio (Matrix), Required<br>5 All of the time<br>4 Most of the time<br>3 More than half of the time<br>2 Less than half of the time<br>1 Some of the time<br>0 At no time
454	who_3_yr	I have felt active and vigorous	radio (Matrix), Required<br>5 All of the time<br>4 Most of the time<br>3 More than half of the time<br>2 Less than half of the time<br>1 Some of the time<br>0 At no time
455	who_4_yr	I woke up feeling fresh and rested	radio (Matrix), Required<br>5 All of the time<br>4 Most of the time<br>3 More than half of the time<br>2 Less than half of the time<br>1 Some of the time<br>0 At no time
456	who_5_yr	My daily life has been filled with things that interest me	radio (Matrix), Required<br>5 All of the time<br>4 Most of the time<br>3 More than half of the time<br>2 Less than half of the time<br>1 Some of the time<br>0 At no time
457	preg_test_yr	Section Header: *The final questions are about any health issues that may be related to your contraception.*<br>Have you had a positive pregnancy test since the last survey?	radio, Required<br>0 No<br>1 Yes<br>88 I don't know
458	pos_preg_date_yr<br>Show the field ONLY if:<br>[preg_test_yr] = '1'	What was the date of your positive pregnancy test?	text (date_mdy)
459	unintended_preg_yr	When you got pregnant, were you trying to get pregnant?	yesno

	Show the field ONLY if: [preg_test_yr] = '1'		1 Yes<br>0 No
460	pos_preg_date_outcome_yr<br>Show the field ONLY if: [preg_test_yr] = '1'	If you have had a positive pregnancy test please describe the outcome of the pregnancy.	radio<br>1 I had or am planning to have an abortion<br>2 I had a miscarriage<br>3 I had an ectopic pregnancy<br>4 I am planning on continuing the pregnancy and keeping the baby<br>5 I am planning on continuing the pregnancy and placing the baby up for adoption<br>88 I am unsure of what I am going to do<br>0 I have not had a positive pregnancy test
461	pos_preg_outcome_date_yr	What was the date when your pregnancy ended, regardless of the outcome?	text (date_mdy)
462	preg_who_where_when_yr<br>Show the field ONLY if: [preg_test_yr] = '1'	To ensure your safety, if you had a pregnancy, we would like to follow-up on the care you received. Please provide the name of the clinic or hospital where you were seen.	notes
463	ae_report_yr	Since enrolling in the study, have you been hospitalized for any illness or injury?	radio, Required<br>0 No<br>1 Yes
464	ae_date_yr<br>Show the field ONLY if: [ae_report_yr] = '1'	Please provide the date:	text (date_mdy), Required
465	ae_description_yr<br>Show the field ONLY if: [ae_report_yr] = '1'	Please explain what happened:	notes, Required
466	iud_se_yr	Have you seen a medical provider for an issue that you thought might be related to the IUD or contraceptive implant you had inserted?	radio, Required<br>0 No<br>1 Yes
467	iud_se_date_yr<br>Show the field ONLY if: [iud_se_yr] = '1'	Please provide the date:	text (date_mdy), Required
468	iud_se_description_yr<br>Show the field ONLY if: [iud_se_yr] = '1'	Please describe:	notes, Required
469	rx_aware_yr	Section Header: *Pharmacy Access*<br>In 2018, Utah passed legislation that allows for pharmacists to dispense birth control pills, patches, and rings to women without a prescription from a doctor.<br><br>Were you aware of this law before now?	yesno<br>1 Yes<br>0 No<br><br>Custom alignment: LH
470	talk_partner_yr	Section Header: *How comfortable do you feel talking about contraception with the following individuals?*<br>Partner(s)	radio (Matrix)<br>1 Very comfortable<br>2 Comfortable<br>3 Neutral<br>4 Uncomfortable<br>5 Very uncomfortable

471	talk_parent_yr	Parent(s) or guardian(s)	radio (Matrix)<br>1 Very comfortable<br>2 Comfortable<br>3 Neutral<br>4 Uncomfortable<br>5 Very uncomfortable
472	talk_friend_yr	Friend(s)	radio (Matrix)<br>1 Very comfortable<br>2 Comfortable<br>3 Neutral<br>4 Uncomfortable<br>5 Very uncomfortable
473	talk_rx_yr	Pharmacist(s)	radio (Matrix)<br>1 Very comfortable<br>2 Comfortable<br>3 Neutral<br>4 Uncomfortable<br>5 Very uncomfortable
474	talk_md_yr	Doctor(s)	radio (Matrix)<br>1 Very comfortable<br>2 Comfortable<br>3 Neutral<br>4 Uncomfortable<br>5 Very uncomfortable
475	talk_teach_yr	Teacher(s)	radio (Matrix)<br>1 Very comfortable<br>2 Comfortable<br>3 Neutral<br>4 Uncomfortable<br>5 Very uncomfortable
476	comfort_yr	Section Header: *Please rate you level of agreement with the following questions.*<br>I am comfortable getting my birth control from a pharmacist without a doctor's prescription.	radio (Matrix)<br>1 Strongly agree<br>2 Agree<br>3 Neutral<br>4 Disagree<br>5 Strongly disagree
477	knowledge_yr	I believe that pharmacist are knowledgeable enough to counsel me on birth control.	radio (Matrix)<br>1 Strongly agree<br>2 Agree<br>3 Neutral<br>4 Disagree<br>5 Strongly disagree
478	toopublic_yr	I worry that my birth control counseling would be too public with a pharmacist.	radio (Matrix)

			1 Strongly agree<br>2 Agree<br>3 Neutral<br>4 Disagree<br>5 Strongly disagree
479	physician_yr	I am more comfortable seeing a physician for birth control.	radio (Matrix)<br>1 Strongly agree<br>2 Agree<br>3 Neutral<br>4 Disagree<br>5 Strongly disagree
480	counsel_yr	I want my pharmacist to counsel me on all of my birth control options.	radio (Matrix)<br>1 Strongly agree<br>2 Agree<br>3 Neutral<br>4 Disagree<br>5 Strongly disagree
481	plan_yr	I plan to get my birth control directly from a pharmacist without seeing a provider first in the next year.	radio (Matrix)<br>1 Strongly agree<br>2 Agree<br>3 Neutral<br>4 Disagree<br>5 Strongly disagree
482	access_yr	Having the option to get birth control from a pharmacist without having to see a provider first will make birth control more accessible to me or someone I know.	radio (Matrix)<br>1 Strongly agree<br>2 Agree<br>3 Neutral<br>4 Disagree<br>5 Strongly disagree
483	rx_reason_no_yr	What are the main reasons that you would not get birth control from a pharmacist?	notes
484	rx_reason_yes_yr	What are the main reasons that you would get birth control from a pharmacist?	notes
485	rx_cost_month_yr	How much would you be willing to pay out-of-pocket for birth control from a pharmacy without having to see a provider first (per month)?<br>*Please enter in X.XX format*	text
486	rx_cost_month_2_yr	How much would you be willing to pay out-of-pocket for a one-time pharmacy consulting fee?<br>*Please enter in X.XX format*	text
487	comments_yr	Section Header: *Thank you again for participating in this study. Feel free to tell us anything about your experience with your contraception or participation in this study.*<br>Comments:	notes
488	brief_followup_survey_1_2_3_years_complete	Section Header: *Form Status*<br>Complete?	dropdown<br>0 Incomplete<br>1 Unverified

			2 Complete

Instrument: **Brief Followup Survey (18 &30 months)** (brief_followup_survey_18_30_months) Enabled as survey Expand

Instrument: **Primer Cuestionario** (primer_cuestionario) Enabled as survey Expand

Instrument: **Cuestionario 1, 3 & 6 Meses** (cuestionario_1_3_6_meses) Enabled as survey Expand

Instrument: **Cuestionario 1, 2 & 3 Años** (cuestionario_1_2_3_aos) Enabled as survey Expand

Instrument: **Cuestionario 18 & 30 Meses** (cuestionario_18_30_meses) Enabled as survey Expand

Instrument: **Covid19 Substudy** (covid19_substudy) Enabled as survey Expand

Instrument: **Covid19 Substudy Spanish** (covid19_substudy_spanish) Enabled as survey Expand
