# Supplementary material for: Changing lives, dynamic plans: Prospective assessment of 12-month changes in pregnancy timing intentions and personal circumstances using data from HER Salt Lake
Source: PLoS One. 2021 Sep 20;16(9):e0257411. doi: 10.1371/journal.pone.0257411 (PMC8451991; doi:10.1371/journal.pone.0257411)
Supplement: S1 Table — (DOCX) [file pone.0257411.s003.docx]

Supplemental Table A: Fixed-Effects Logistic Regression Models of Changing from Never Desiring a Pregnancy to Considering a Pregnancy in the future at 12-month Follow-up (Adjusted Odds Ratios shown)

|  | *Model 1* | Model 2 | Model 3 | Model 4 | Model 4 |
| --- | --- | --- | --- | --- | --- |
|  | aOR (95% Confidence Interval) | aOR (95% Confidence Interval) | aOR (95% Confidence Interval) | aOR (95% Confidence Interval) | aOR (95% Confidence Interval) |
| *Relationship status.* |  |  |  |  |  |
| Single (or divorce), not dating | reference | reference | reference | reference |  |
| Married | 5.24 (0.84 - 32.53) | 4.95 (0.93 - 26.27) | 3.11 (0.86 - 11.29) | 5.92 (1.32 - 26.51) | 5.35 (0.35 - 82.76) |
| Living together with a partner | 2.74 (1.29 - 5.81) | 2.63 (1.42 - 4.88) | 2.75 (1.51 - 5.03) | 3.25 (1.70 - 6.23) | 3.14 (1.30 - 7.58) |
| Actively dating | 0.51 (0.23 - 1.14) | 0.44 (0.21 - 0.91) | 0.47 (0.22 - 0.97) | 0.44 (0.21 - 0.92) | 0.68 (0.27 - 1.72) |
|  |  |  |  |  |  |
| *Sexual identity*. |  |  |  |  |  |
| Exclusively heterosexual | reference | reference | reference | reference |  |
| Mostly heterosexual | 3.50 (1.17 - 10.52) | 1.79 (0.71 - 4.51) | 1.91 (0.83 - 4.39) | 2.22 (0.89 - 5.52) | 1.88 (0.46 - 7.58) |
| Mostly or exclusively gay/lesbian, bisexual, or other sexuality | 2.83 (0.56 - 14.31) | 1.43 (0.44 - 4.69) | 2.04 (0.71 - 5.85) | 2.52 (0.81 - 7.88) | 1.24 (0.16 - 9.43) |
|  |  |  |  |  |  |
| *Educational enrollment and goals* |  |  |  |  |  |
| Enrolled full-time | reference |  |  |  |  |
| Not enrolled, no further goals | 1.68 (0.45 - 6.28) |  |  |  | 1.95 (0.37-10.17) |
| Not enrolled, has goals for further education | 2.22 (0.81 - 6.12) |  |  |  | 2.87 (0.75-11.00) |
| Enrolled part-time | 1.98 (0.66 - 5.92) |  |  |  | 1.97 (0.49- 7.90) |
|  |  |  |  |  |  |
| *Household income* *(as % of federal poverty level,* in 10% increments) |  | 1.00 (1.00 - 1.00) |  |  | 1.06 (1.00 - 1.13) |
| Currently has *health insurance* |  |  | 2.32 (1.29 - 4.17) |  | 1.17 (0.49 - 2.83) |
| *Employment status.* |  |  |  |  |  |
| Unemployed |  |  |  | reference |  |
| Employed full-time |  |  |  | 5.81 (2.12 - 15.92) | 5.94 (1.29 - 27.36) |
| Employed part-time |  |  |  | 2.15 (0.71 - 6.55) | 1.20 (0.23 - 6.23) |
| Other Employment Status (homemaker, disability, etc.) |  |  |  | 3.12 (1.07 - 9.08) | 3.47 (0.66 - 18.13) |
|  |  |  |  |  |  |
| Observations | 276 | 326 | 388 | 378 | 238 |
| Number of Participants | 138 | 163 | 194 | 189 | 119 |

Note: We present fixed effects models, which include only participants for whom outcomes have changed between enrollment and 12-month follow up. 208 participants reported changed from never desiring a pregnancy to considering a pregnancy at 12-month follow-up. Analytic sample sizes vary across models due to missing data on included covariates, which vary across the four models. Separate models are used to illustrate the effects of the key covariate without potentially overcontrolling (Models 1-4). The model containing all covariates shown here is shown in Table 3 in the body of the paper.

Supplemental Table B: Fixed-Effects Logistic Regression Models of Desiring a Pregnancy Now or in the Next Year (Adjusted Odds Ratios shown)

|  | Model 1 |  | Model 2 |  | | Model 3 |  | Model 4 |  | Model 5 |  |
| --- | --- | --- | --- | --- | --- | --- | --- | --- | --- | --- | --- |
|  | aOR (95% Confidence Interval) | | aOR (95% Confidence Interval) | | | aOR (95% Confidence Interval) | | aOR (95% Confidence Interval) | | aOR (95% Confidence Interval) | |
| Relationship status. | | |  |  | |  |  |  |  |  |  |
| Single (or divorce), not dating | reference | | reference | | | reference | | reference | | reference | |
| Married | 10.81  (3.01 - 38.91) | | 27.03 (5.64 - 129.51) | | | 12.97 (3.86 - 43.53) | | 20.32 (5.78 - 71.37) | | 80.93 (3.86 - 1,697.02) | |
| Living together with a partner | 1.69  (0.72 - 3.96) | | 2.66 (1.06 - 6.67) | | | 1.75 (0.80 - 3.83) | | 2.23 (1.04 - 4.80) | | 4.78 (0.91 - 25.23) | |
| Actively dating | 0.20  (0.05 - 0.93) | | 0.42 (0.10 - 1.82) | | | 0.37 (0.10 - 1.34) | | 0.24 (0.06 - 0.95) | | 0.54 (0.05 - 5.43) | |
|  |  |  |  |  | |  |  |  |  |  |  |
| Sexual identity. | |  |  |  | |  |  |  |  |  |  |
| Exclusively heterosexual | reference | | reference | | reference | | | reference | | reference | |
| Mostly heterosexual | 3.68 (0.77 - 17.52) | | 5.24 (1.04 - 26.32) | | | 4.03  (1.24 - 13.09) | | 3.39 (1.01 - 11.44) | | 265.16 (1.59 - 44,239.40) | |
| Mostly or exclusively gay/lesbian, bisexual, or other sexuality | 4.19 (0.51 - 34.44) | | 26.82 (1.23 - 582.64) | | | 2.13 (0.45 - 10.14) | | 2.40 (0.44 - 13.09) | | 4,387.73 (2.87 - 6,709,706.94) | |
|  |  |  |  |  | |  |  |  |  |  |  |
| Educational enrollment and goals | | | | | |  |  |  |  |  |  |
| Enrolled full-time | reference | |  |  | |  |  |  |  | reference | |
| Not enrolled, no further goals | 5.33 (0.95 - 29.78) | |  |  | |  |  |  |  | 19.82 (0.88 - 446.40) | |
| Not enrolled, has goals for further education | 8.74 (1.87 - 40.83) | |  |  | |  |  |  |  | 12.75 (0.77 - 211.32) | |
| Enrolled part-time | 5.54 (1.16 - 26.32) | |  |  | |  |  |  |  | 19.73 (1.16 - 336.12) | |
|  |  |  |  |  | |  |  |  |  |  |  |
| Household income (as % of federal poverty level, in 10% increments) | | | 1.13 (1.07 - 1.19) | | |  |  |  |  | 1.27 (1.10 - 1.47) | |
| Currently has health insurance | | | |  | | 5.19 (2.54 - 10.61) | |  |  | 4.96 (1.11 - 22.19) | |
| Employment status. | | |  |  | |  |  |  |  |  |  |
| Unemployed | |  |  |  | |  |  |  |  |  |  |
| Employed full-time | | |  |  | |  |  | 2.61 (1.10 - 6.19) | | 2.42 (0.30 - 19.49) | |
| Employed part-time | | |  |  | |  |  | 0.88 (0.27 - 2.92) | | 2.20 (0.11 - 45.96) | |
| Other Employment Status (homemaker, disability, etc.) | | | | | | | | 0.91 (0.32 - 2.56) | | 0.30 (0.04 - 2.46) | |
|  |  |  |  |  | |  |  |  |  |  |  |
| Observations | 282 | | 344 | | | 418 | | 398 | | 236 | |
| Number of Participants | 141 | | 172 | | | 209 | | 199 | | 118 | |

Note: We present non-nested fixed effects models, which include only participants for whom the outcome (desiring a pregnancy now or in the next 12 months) has changed between enrollment and 12-month follow up. Analytic sample sizes vary across models due to missing data on included covariates, which vary across the four models. Separate models are used to illustrate the effects of the key covariate without potentially overcontrolling (Models 1-4). Model 5 includes all of the key contextual measures, but due to the extreme size in the odds ratios for sexual identity (likely due to very small cell sizes), effects should only be interpreted with caution.

Supplemental Table C: Individual Characteristics of Participants who Indicated with an Unintended Pregnancy between the two Waves or who had a positive pregnancy test with unclear intendedness

|  | | | Obs. | Mean/Proportion | St. Dev. | Min. | Max. |
| --- | --- | --- | --- | --- | --- | --- | --- |
| *Age at enrollment (in years)*^1^ | | | 76 | 24.55 | 5.71 | 18.31 | 42.01 |
|  |  |  |  |  |  |  |  |
| *Race/Ethnicity^1^* | |  |  |  |  |  |  |
| White, non-Hispanic | |  | 76 | 0.57 |  | 0 | 1 |
| Hispanic (any racial group) | | | 76 | 0.32 |  | 0 | 1 |
| Non-white, non-Hispanic/Latinx | | | 76 | 0.12 |  | 0 | 1 |
| *Sexual identity* | |  |  |  |  |  |  |
| Exclusively heterosexual | | | 73 | 0.59 |  | 0 | 1 |
| Mostly heterosexual | |  | 73 | 0.19 |  | 0 | 1 |
| Mostly or exclusively gay/lesbian, bisexual, or other sexuality | | | 73 | 0.22 |  | 0 | 1 |
| *Relationship status* | |  |  |  |  |  |  |
| Married |  |  | 76 | 0.22 |  | 0 | 1 |
| Living together with a partner | | | 76 | 0.55 |  | 0 | 1 |
| Actively dating | |  | 76 | 0.08 |  | 0 | 1 |
| Single (or divorced), not dating | | | 76 | 0.14 |  | 0 | 1 |
| *Educational enrollment and goals* | | | |  |  |  |  |
| Not enrolled, no further goals | | | 58 | 0.22 |  | 0 | 1 |
| Not enrolled, has goals for further education | | | 58 | 0.47 |  | 0 | 1 |
| Enrolled part-time | |  | 58 | 0.14 |  | 0 | 1 |
| Enrolled full-time | |  | 58 | 0.17 |  | 0 | 1 |
| *Household income (as % of federal poverty level)* | |  | 67 | 175.81 | 136.90 | 13.61 | 530.59 |
| *Health insurance* | |  |  |  |  |  |  |
| Has any form of health insurance | |  | 76 | 0.75 |  | 0 | 1 |
| No health insurance | |  | 76 | 0.25 |  | 0 | 1 |
| *Employment status* | |  |  |  |  |  |  |
| Unemployed | |  | 76 | 0.18 |  | 0 | 1 |
| Employed full-time | |  | 76 | 0.51 |  | 0 | 1 |
| Employed part-time | |  | 76 | 0.13 |  | 0 | 1 |
| Other (homemaker, student, disabled, sick leave, other) |  |  | 76 | 0.17 |  | 0 | 1 |
